# High rate of detected variants in male *PLCZ1* and *ACTL7A* genes causing failed fertilization after ICSI

**DOI:** 10.1093/hropen/hoae057

**Published:** 2024-09-28

**Authors:** Arantxa Cardona Barberán, Ramesh Reddy Guggilla, Cora Colenbier, Emma Van der Velden, Andrei Rybouchkin, Dominic Stoop, Luc Leybaert, Paul Coucke, Sofie Symoens, Annekatrien Boel, Frauke Vanden Meerschaut, Björn Heindryckx

**Affiliations:** Ghent-Fertility And Stem cell Team (G-FaST), Department for Reproductive Medicine, Ghent University Hospital, Ghent, Belgium; Ghent-Fertility And Stem cell Team (G-FaST), Department for Reproductive Medicine, Ghent University Hospital, Ghent, Belgium; MedGenome Labs Ltd, Narayana Health City, Bengaluru, India; Ghent-Fertility And Stem cell Team (G-FaST), Department for Reproductive Medicine, Ghent University Hospital, Ghent, Belgium; Ghent-Fertility And Stem cell Team (G-FaST), Department for Reproductive Medicine, Ghent University Hospital, Ghent, Belgium; Ghent-Fertility And Stem cell Team (G-FaST), Department for Reproductive Medicine, Ghent University Hospital, Ghent, Belgium; Ghent-Fertility And Stem cell Team (G-FaST), Department for Reproductive Medicine, Ghent University Hospital, Ghent, Belgium; Physiology Group, Department of Basic and Applied Medical Sciences, Ghent University, Ghent, Belgium; Department of Biomolecular Medicine, Center for Medical Genetics Ghent (CMGG), Ghent University Hospital, Ghent, Belgium; Department of Biomolecular Medicine, Center for Medical Genetics Ghent (CMGG), Ghent University Hospital, Ghent, Belgium; Ghent-Fertility And Stem cell Team (G-FaST), Department for Reproductive Medicine, Ghent University Hospital, Ghent, Belgium; Ghent-Fertility And Stem cell Team (G-FaST), Department for Reproductive Medicine, Ghent University Hospital, Ghent, Belgium; Ghent-Fertility And Stem cell Team (G-FaST), Department for Reproductive Medicine, Ghent University Hospital, Ghent, Belgium

**Keywords:** male infertility, fertilization failure after ICSI, *PLCZ1* variants, *ACTL7A* variants, assisted oocyte activation, calcium oscillation pattern

## Abstract

**STUDY QUESTION:**

What is the frequency of *PLCZ1*, *ACTL7A*, and *ACTL9* variants in male patients showing fertilization failure after ICSI, and how effective is assisted oocyte activation (AOA) for them?

**SUMMARY ANSWER:**

Male patients with fertilization failure after ICSI manifest variants in *PLCZ1* (29.09%), *ACTL7A* (14.81%), and *ACTL9* (3.70%), which can be efficiently overcome by AOA treatment with ionomycin.

**WHAT IS KNOWN ALREADY:**

Genetic variants in *PLCZ1*, and more recently, in *ACTL7A*, and *ACTL9* male genes, have been associated with total fertilization failure or low fertilization after ICSI. A larger patient cohort is required to understand the frequency at which these variants occur, and to assess their effect on the calcium ion (Ca^2+^) release during oocyte activation. AOA, using ionomycin, can restore fertilization and pregnancy rates in patients with *PLCZ1* variants, but it remains unknown how efficient this is for patients with *ACTL7A* and *ACTL9* variants.

**STUDY DESIGN, SIZE, DURATION:**

This prospective study involved two patient cohorts. In the first setting, group 1 (N = 28, 2006–2020) underwent only *PLCZ1* genetic screening, while group 2 (N = 27, 2020–2023) underwent *PLCZ1, ACTL7A*, and *ACTL9* genetic screening. Patients were only recruited when they had a mean fertilization rate of ≤33.33% in at least one ICSI cycle with at least four MII oocytes. Patients underwent a mouse oocyte activation test (MOAT) and at least one ICSI–AOA cycle using calcium chloride (CaCl_2_) injection and double ionomycin exposure at our centre. All patients donated a saliva sample for genetic screening and a sperm sample for further diagnostic tests, including Ca^2+^ imaging.

**PARTICIPANTS/MATERIALS, SETTING, METHODS:**

Genetic screening was performed via targeted next-generation sequencing. Identified variants were classified by applying the revised ACMG guidelines into a Bayesian framework and were confirmed by bidirectional Sanger sequencing. If variants of uncertain significance or likely pathogenic or pathogenic variants were found, patients underwent additional determination of the sperm Ca^2+^-releasing pattern in mouse (MOCA) and in IVM human (HOCA) oocytes. Additionally, ACTL7A immunofluorescence and acrosome ultrastructure analyses by transmission electron microscopy (TEM) were performed for patients with *ACTL7A* and/or *ACTL9* variants.

**MAIN RESULTS AND THE ROLE OF CHANCE:**

Overall, the frequency rate of *PLCZ1* variants was 29.09%. Moreover, 14.81% of patients carried *ACTL7A* variants and 3.70% carried *ACTL9* variants. Seven different *PLCZ1* variants were identified (p.Ile74Thr, p.Gln94*, p.Arg141His, p.His233Leu, p.Lys322*, p.Ile379Thr, and p.Ser500Leu), five of which are novel. Interestingly, *PLCZ1* variants p.Ser500Leu and p.His233Leu occurred in 14.55% and 9.09% of cases. Five different variants were found in *ACTL7A* (p.Tyr183His, p.Gly214Ser, p.Val340Met, p.Ser364Glnfs*9, p.Arg373Cys), four of them being identified for the first time. A novel variant in *ACTL9* (p.Arg271Pro) was also described. Notably, both heterozygous and homozygous variants were identified.

The MOCA and HOCA tests revealed abnormal or absent Ca^2+^ release during fertilization in all except one patient, including patients with *PLCZ1* heterozygous variants. TEM analysis revealed abnormal acrosome ultrastructure in three patients with *ACTL7A* variants, but only patients with homozygous *ACTL7A* variants showed reduced fluorescence intensity in comparison to the control.

AOA treatment significantly increased the fertilization rate in the 19 patients with detected variants (from 11.24% after conventional ICSI to 61.80% after ICSI–AOA), as well as positive hCG rate (from 10.64% to 60.00%) and live birth rate (from 6.38% to 37.14%), resulting in 13 healthy newborns. In particular, four live births and two ongoing pregnancies were produced using sperm from patients with *ACTL7A* variants.

**LIMITATIONS, REASONS FOR CAUTION:**

Genetic screening included exonic and outflanking intronic regions, which implies that deep intronic variants were missed. In addition, other male genes or possible female-related factors affecting the fertilization process remain to be investigated.

**WIDER IMPLICATIONS OF THE FINDINGS:**

Genetic screening of *PLCZ1*, *ACTL7A*, and *ACTL9* offers a fast, cost-efficient, and easily implementable diagnostic test for total fertilization failure or low fertilization after ICSI, eliminating the need for complex diagnostic tests like MOAT or Ca^2+^ analysis. Nonetheless, HOCA remains the most sensitive functional test to reveal causality of uncertain significance variants. Interestingly, heterozygous *PLCZ1* variants are sufficient to cause inadequate Ca^2+^ release during ICSI. Most importantly, AOA treatment using CaCl_2_ injection followed by double ionomycin exposure is highly effective for this patient group, including those with *ACTL7A* variants, who also display a Ca^2+^-release deficiency.

**STUDY FUNDING/COMPETING INTEREST(S):**

This study was supported by the Flemish Fund for Scientific Research (FWO) (TBM-project grant T002223N awarded to B.H.) and by the Special Research Fund (BOF) (starting grant BOF.STG.2021.0042.01 awarded to B.H.). A.C.B., R.R.G., C.C., E.V.D.V., A.R., D.S., L.L., P.C., S.S., A.B., and F.V.M. have nothing to disclose. B.H. reports a research grant from FWO and BOF, and reports being a board member of the Belgian Ethical Committee on embryo research.

**TRIAL REGISTRATION NUMBER:**

N/A.

WHAT DOES THIS MEAN FOR PATIENTS? This research aims to understand why some couples experience fertilization failure (FF) after ICSI. Specifically, this study investigates the occurrence of mutations in three male genes (*PLCZ1*, *ACTL7A*, and *ACTL9*) in patients who previously had recurrent fertilization rates ≤33.33% after ICSI and evaluates the effectiveness of assisted oocyte activation (AOA) treatment in overcoming this infertility problem. Normal fertilization requires the generation of calcium oscillations in the oocyte. AOA treatment mimics this process by using chemicals, like ionomycin, to artificially induce calcium release in the oocytes.

Through genetic testing, this study found *PLCZ1*, *ACTL7A*, and *ACTL9* mutations in 29.09%, 14.81%, and 3.70% of cases, respectively. These specific genetic variations were associated with abnormal calcium activity during fertilization. AOA treatment using ionomycin was highly effective in improving fertilization rates for all patients with these mutations. In conclusion, genetic screening of men for these genes could help IVF laboratories provide better patient counselling and personalized treatment strategies for couples dealing with FF after ICSI.

## Introduction

While ICSI is highly effective in overcoming male infertility (Palermo *et al.*, 2017), 1–3% of ICSI cycles result in total fertilization failure (FF) (Flaherty *et al.*, 1998; Kashir *et al.*, 2010; Bhattacharya *et al.*, 2013). Oocyte activation failure has been associated with absent, reduced, or abnormal phospholipase C zeta (PLCζ) protein content in sperm cells (Yoon *et al.*, 2008; Cheung *et al.*, 2020; Yan *et al.*, 2020). PLCζ protein, recognized as the sperm-born oocyte activation factor, is responsible for inducing recurrent cytoplasmic calcium ion (Ca^2+^) oscillations in the oocytes after sperm–oocyte fusion or intracytoplasmic sperm injection (Kashir *et al.*, 2010; Nomikos *et al.*, 2013; Hachem *et al.*, 2017). These Ca^2+^ oscillations are essential for multiple processes required for the completion of fertilization, including the prevention of polyspermy, the culmination of meiosis, and the formation of pronuclei. Currently, about 30 different *PLCZ1* variants have been identified in male patients suffering from total FF or low fertilization after ICSI (hereafter described as FF after ICSI) (Yoon *et al.*, 2008; Heytens *et al.*, 2009; Escoffier *et al.*, 2016; Torra-Massana *et al.*, 2019; Dai *et al.*, 2020; Mu *et al.*, 2020; Wang *et al.*, 2020; Yan *et al.*, 2020; Yuan *et al.*, 2020a,b; Lin *et al.*, 2023; Peng *et al.*, 2023). However, these data differ in terms of inclusion criteria (number of ICSI cycles and mean fertilization rate), genetic ancestry group, number of patients screened and, consequently, the observed variant detection rate. *PLCZ1* variants have been reported in one-third of the patients analysed so far (Torra-Massana *et al.*, 2019; Dai *et al.*, 2020; Yan *et al.*, 2020). In addition, all reports have identified homozygous or compound heterozygous *PLCZ1* variants, except one which reported heterozygous variants (Torra-Massana *et al.*, 2019) as the sole cause of oocyte activation failure due to haploinsufficiency. Clearly, more extensive evidence is needed to confirm whether heterozygous *PLCZ1* variants suffice to impair fertilization after ICSI. Overall, there is a lack of functional testing of the reported variants at the level of oocyte Ca^2+^-release responses and consequent fertilization potential.

Recently, actin-like protein 7A (*ACTL7A*) and 9 (*ACTL9*) variants have also been associated with FF and embryo developmental arrest after ICSI (Xin *et al.*, 2020; Dai *et al.*, 2021). Both ACTL7A and ACTL9 play a role in the formation and fusion of Golgi-derived vesicles during acrosome biogenesis (Xin *et al.*, 2020; Dai *et al.*, 2021; Zhou *et al.*, 2022). These proteins, located in the sub-acrosomal layer of the perinuclear theca (PT) within the sperm head (Zhou *et al.*, 2022; Moreno, 2023), are crucial for maintaining acrosome attachment to the nuclear membrane. Defects in ACTL7A or ACTL9 can lead to acrosome detachment, enlarging the PT. A swollen PT appears to disturb PLCζ localization and content, which explains the low fertilization rates observed in these patients (Wang *et al.*, 2021). Thus far, nine different *ACTL7A* variants (Xin *et al.*, 2020; Wang *et al.*, 2021; Chen *et al.*, 2022; Dai *et al.*, 2022a; Zhao *et al.*, 2023; Zhou *et al.*, 2023) and three *ACTL9* variants (Dai *et al.*, 2021) have been reported in patients with FF after ICSI, but larger cohort studies with clear inclusion criteria are required to understand the frequency rate at which these variants occur.

Application of assisted oocyte activation (AOA) using Ca^2+^ ionophores during ICSI has been shown to correct the fertilization problem to attain live births at great efficiency in patients with FF after ICSI (Bonte *et al.*, 2019; Cardona Barberán *et al.*, 2022), notably when using ionomycin instead of calcimycin (Nikiforaki *et al.*, 2016; Jia *et al.*, 2023). For patients with *PLCZ1* variants, in which there is a clear Ca^2+^-release deficiency, AOA is highly effective (Torra-Massana *et al.*, 2019; Dai *et al.*, 2022a). However, for patients with *ACTL7A* and *ACTL9* variants, AOA efficacy is not proven yet (Xin *et al.*, 2020; Zhou *et al.*, 2023). AOA treatment, using in-house prepared calcimycin, has been applied in patients with *ACTL7A* variants, and although fertilization increased compared to previous ICSI cycles, only two live births have been achieved so far (Wang *et al.*, 2021; Dai *et al.*, 2022a; Zhao *et al.*, 2023). Currently, it is unknown whether sperm from patients with *ACTL7A* and *ACTL9* variants trigger abnormal Ca^2+^ release during fertilization, for which AOA treatment would be beneficial.

In this study, a large patient cohort suffering from FF after ICSI was recruited to investigate the frequency of *PLCZ1, ACTL7A*, and *ACTL9* variants. To assess the pathogenicity of the identified variants on the PLCζ protein content, the Ca^2+^ oscillation pattern after patient sperm injection into mouse and human oocytes was evaluated. To assess the pathogenicity of the identified *ACTL7A* and *ACTL9* variants, immunofluorescence analysis as well as acrosome ultrastructure evaluation by transmission electron microscopy were conducted. Most importantly, AOA efficiency was evaluated for all patients with detected variants using our standard AOA protocol (Heindryckx *et al.*, 2005, 2008; Bonte *et al.*, 2019).

## Materials and methods

### Ethical approval

Approval was obtained from the Ghent University Hospital Ethical Committee for this study (EC 2010/808 and EC 2014/1309). All patients provided informed consent to donate saliva and sperm samples for this research. They also agreed to undergo the mouse oocyte activation test (MOAT) and an ICSI–AOA cycle, a routine procedure in our IVF clinic for patients experiencing FF after ICSI. Approval for assays involving laboratory animals (MOAT and mouse oocyte calcium analysis (MOCA)) was granted by the Ghent University Hospital Ethical Committee for Laboratory Animals (ECD 19/66 and 19/67).

### Study design, setting, and participants

This study involved two patient cohorts with a total of 55 patients. Cohort 1 comprising 28 male patients (P1–P28) recruited from 2006 to 2020, underwent solely *PLCZ1* genetic screening, while cohort 2, consisting of 27 male patients (P29–P55) recruited from 2020 to 2023 was subjected to *PLCZ1, ACTL7A*, and *ACTL9* genetic screening. *ACTL7A* and *ACTL9* genes were not screened in cohort 1 because at that time, mutations in these genes were not yet discovered, and afterwards, there were insufficient saliva samples remaining. Inclusion criteria required total FF or low fertilization (mean fertilization rate ≤33.33%) in one or more ICSI cycles with at least four metaphase II (MII) oocytes injected (Supplementary Table S1). Many patients were referred from external IVF clinics, some having undergone an ICSI–AOA cycle with limited efficacy (Supplementary Table S2). Patients underwent the MOAT (Supplementary Table S3) and at least one ICSI–AOA cycle at the Ghent University Hospital (Supplementary Table S4). Exclusion criteria included severe male factor (sperm concentration <0.1 M/ml) or globozoospermia (Supplementary Table S3). The mean maternal age was 33.06 ± 4.26 years in the last ICSI cycle and 34.62 ± 4.07 in the last ICSI-AOA cycle. Patients in whom variants of uncertain significance (VUS) or likely pathogenic or pathogenic variants were identified donated a sperm sample for functional testing.

### Sperm sample collection and MOAT

Sperm concentration, motility, and morphology were evaluated following World Health Organization guidelines (WHO, 2021) (Supplementary Table S3) before freezing with SpermFreeze™ (FertiPro, Beernem, Belgium). The patients’ sperm capacity to activate mouse oocytes was analysed using the MOAT as previously described by Heindryckx *et al.* (2008) (Supplementary Table S3). Thawed sperm cells were injected into MII mouse oocytes using PIEZO-ICSI (Yoshida and Perry, 2007) on a cooled stage (15–17°C). Injected oocytes were cultured in potassium simplex optimized medium (KSOM) supplemented with 0.4% bovine serum albumin (BSA, Merck Life Science, Hoeilaart, Belgium) (KSOM-BSA) at 37°C under standard conditions (6% CO_2_, 5% O_2_, and 89% N_2_), and the activation rate (2-cell embryos/MII oocytes) was assessed 24 h later. Patients were classified as follows: MOAT 1 (activation rate ≤20%), MOAT 2 (activation rate between 21% and 84%), or MOAT 3 (activation rate >85%).

### Genetic screening

Patient saliva samples were collected in Oragene 0G-500 kits (DNA Genotek, Ottawa, Canada) and processed for genomic DNA as per the manufacturer’s instructions. Quantification was performed using the Qubit™ dsDNA HS Assay Kit (Thermo Fisher Scientific, Waltham, MA, USA). The coding exons and adjacent intronic regions of *PLCZ1* (NM_033123.4), *ACTL7A* (NM_006687.4), and *ACTL9* (NM_178525.5) genes were amplified through singleplex PCR (Lefever *et al.*, 2017) and subjected to targeted next-generation sequencing (MiSeq, Illumina, San Diego, CA, USA) at the Center for Medical Genetics Ghent (De Leeneer *et al.*, 2015). Primers used for genetic screening are available upon request.

### Variant classification

Identified variants were classified according to the revised American College of Medical Genetics guidelines within a Bayesian framework (Richards *et al.*, 2015; Tavtigian *et al.*, 2018) by two operators and using an in-house classification tool. The benign and pathogenic classification criteria selected for each variant are detailed in Supplementary Table S5. The ClinGen Bayesian classification system allocates variants to benign, likely benign, uncertain significance, likely pathogenic and pathogenic groups, and provides a posterior probability score (*P*) which indicates the likelihood of the variant to favour a more benign or pathogenic interpretation.

Variants of interest were filtered when allele frequency was <5% in gnomAD v4.0.0 (https://gnomad.broadinstitute.org). This high-frequency threshold was selected as FF after ICSI is a rare condition for which disease penetrance and prevalence are not well established. Amino acid conservation was examined using UCSC Genome Browser (https://genome-euro.ucsc.edu/). Pathogenicity was assessed using the *in silico* prediction REVEL (rare exome variant ensemble learner) meta score (Ioannidis *et al.*, 2016), that integrates the predictions from several individual tools, including MutPred, FATHMM, VEST, PolyPhen, SIFT, PROVEAN, MutationAssessor, MutationTaster, LRT, GERP, SiPhy, phyloP, and phastCons. Predicted three-dimensional protein structure models of the wild-type PLCZ1 (AF-Q86YW0-F1), ACTL7A (AF-Q9Y615-F1), and ACTL9 (AF-Q8TC94-F1) proteins obtained from AlphaFold (https://alphafold.ebi.ac.uk/) were used to investigate the effect of both protein-truncating and missense variants on protein conformation using AlphaMissense (Cheng *et al.*, 2023) and Missense3D (Ittisoponpisan *et al.*, 2019) (Supplementary Fig. S1). Final protein structure figures were prepared with UCSF Chimera (Pettersen *et al.*, 2004).

Segregation studies were not possible as parental DNA samples were not available, except for P44. VUS, or likely pathogenic or pathogenic variants were confirmed by bidirectional Sanger sequencing (ABI3730XL, Applied Biosystems, Foster City, CA, USA) (Supplementary Fig. S2).

### Mouse and human oocyte collection

MII mouse oocytes were collected from 8- to 12-week-old B6D2F1 female mice (Janvier Labs, Le Genest-Saint-Isle, France) after PMSG (Folligon, MSD AH, Brussels, Belgium) and hCG (Chorulon, MSD AH, Brussels, Belgium) injections. At 12–14 h after hCG, cumulus–oocyte complexes were denuded with hyaluronidase and cultured in KSOM–BSA at standard conditions until further manipulations (MOAT and MOCA) (Vanden Meerschaut *et al.*, 2013).

Spare immature human oocytes donated from IVF cycles at Ghent University Hospital were used for research after written informed consent was given. Germinal vesicle (GV) oocytes were cultured for 24 h in IVM medium as described by Christodoulaki *et al.* (2023), while metaphase I (MI) oocytes were cultured for either 3 or 24 h in Sydney IVF Cleavage Medium (Cook Medical, Limerick, Ireland). Successfully *in vitro* matured MII oocytes were used for human oocyte calcium analysis (HOCA).

### MOCA and HOCA

To assess the calcium-releasing capacity of sperm with identified variants, we conducted the MOCA and HOCA as previously described (Vanden Meerschaut *et al.*, 2013; Ferrer-Buitrago *et al.*, 2018). MII mouse or human oocytes were exposed to 7.5 µM Ca^2+^-sensitive fluorescent dye Fura-2 AM (Merck Life Science, Hoeilaart, Belgium) or Fura-2 LR/AM (Merck Life Science), respectively. The injected oocytes were imaged for 2 h (MOCA) or for 10 h (HOCA) with an inverted epifluorescence microscope. Data were analysed using the Clampfit 10.7 software (Molecular Devices LLC, San Jose, CA, USA) and the total Ca^2+^ released was calculated as the product of the mean amplitude (A) and mean frequency (F) (A×F) of Ca^2+^ oscillations over time. In addition, Ca^2+^ spike frequencies were categorized as follows: (0) absence of oscillations, (+) 1–2 oscillations, (++) 3–9 oscillations, (+++) 10–20 oscillations, or (++++) >20 oscillations. Representative graphs are shown in Supplementary Fig. S3.

### Immunofluorescence staining

Thawed sperm from patients with *ACTL7A* variants were fixed in 4% paraformaldehyde (PFA) (Merck Life Science) for 30 min at RT, centrifuged (493 *g*, 5 min) and resuspended in 1× PBS. Samples with at least 10^6^ sperm cells/ml were applied onto pre-coated multiwell slides (18357-1, Polyscences, Hirschberg an der Bergstrasse, Germany), permeabilized with 1% Triton X-100 (Merck Life Science) for 30 min at RT and then blocked in 1× PBS, 4% BSA for 30 min at RT. Afterwards, sperm cells were incubated with rabbit polyclonal anti-ACTL7A antibody (1:100, HPA021624, Atlas Antibodies, Stockholm, Sweden) overnight at 4°C. For negative control samples, 1× PBS, 1% BSA was applied instead of primary antibody. Subsequently, secondary donkey anti-rabbit IgG antibody (1:750, Alexa Fluor^®^ 594, Ab150076, Abcam, Waltham, MA, USA) was applied for 1 h at 37°C followed by counterstaining with Hoechst 33258 (1:100, 94403, Merck Life Science) and Lectin from *Pisum sativum* FITC conjugate (1:50, PSA-FITC, L0770, Merck Life Science) for 30 min at 37°C. Samples were washed in 1× PBS or 1× PBS, 1% BSA after each step. Finally, slides were mounted in Mowiol-PPD and imaged using a confocal laser scanning microscope (LSM 900; Zeiss, Zaventem, Belgium) at 40 × 2.5× magnification. At least 50 sperm cells with intact acrosomes (Supplementary Fig. S4) were captured in multiple fields. The fluorescence intensity of ACTL7A in sperm heads was analysed using Fiji (Schindelin *et al.*, 2012). The proportion of sperm cells with invisible, weak, or obvious ACTL7A expression was evaluated considering the mean grey value (fluorescence intensity) in the sperm head after background correction.

### Transmission electron microscopy

Thawed sperm from patients with *ACTL7A* or *ACTL9* variants and healthy control were fixed in 4% PFA and 2.5% glutaraldehyde in 0.1 M Na cacodylate buffer, pH 7.2, and centrifuged at 277 *g*. Low melting point-agarose was used to keep the cells concentrated for further processing. Cells were fixed for 4 h at RT followed by fixation overnight at 4°C after replacing with fresh fixative. After washing, the cells were post-fixed in 1% OsO_4_ with 1.5% K_3_Fe(CN)_6_ in 0.1 M Na cacodylate buffer at RT for 1 h, dehydrated through a graded ethanol series, bulk stained with 1% uranyl acetate at the 50% ethanol step, and embedded in Spurr’s resin. Ultrathin sections of a gold interference colour were cut using an ultra-microtome (Leica EM UC6; Leica Biosystems, Nussloch, Germany), followed by a post-staining in a Leica EM AC20 for 40 min in uranyl acetate at 20°C and for 10 min in lead stain at 20°C. Sections were collected on Formvar-coated copper slot grids. Grids were viewed with a JEM-1400plus transmission electron microscope (JEOL, Tokyo, Japan) operating at 80 kV and 10 000× magnification. At least 20 sperm heads with intact acrosomes (Supplementary Fig. S4B) were included in the analysis. Acrosomes were considered detached from the nuclear envelope when the distance of detachment was ≥50% of the size of the acrosome or showed a clear folding and curved shape, with >50% of the observed acrosome detached (Supplementary Fig. S4B).

### ICSI and ICSI–AOA

For ICSI and ICSI–AOA cycles, female partners were stimulated with either a GnRH agonist or an antagonist protocol, as detailed in [Bibr hoae057-B20][Bibr hoae057-B20]. Oocytes were retrieved after 36-h post-hCG administration, decumulated with hyaluronidase (ICSI Cumulase, CooperSurgical, Ballerup, Denmark), and cultured in Sydney IVF Fertilization Medium (Cook Medical, Limerick, Ireland) until further sperm injection by standard ICSI. The AOA protocol was performed as previously described (Rybouchkin *et al.*, 1997; Heindryckx *et al.*, 2008). Briefly, patient sperm was injected into MII oocytes together with 0.1 M CaCl_2_, followed by two 10-min exposures to 10 μM ionomycin (I9657, Merck Life Science, Hoeilaart, Belgium) with a 30-min interval. After extensive washing, oocytes were cultured in Sydney IVF Cleavage Medium (Cook Medical) until Day 3 (D3) and in Sydney IVF Blastocyst Medium (Cook Medical) from D3 to D5 under standard conditions. Embryo transfers were performed on D2 or D3 in four cycles carried out before 2012, while in the rest of the cycles, the transfer was on D5. Embryo classification was performed as described in Cardona Barberán *et al.* (2023).

### Statistics

Statistical analysis was performed using GraphPad Prism version 10.0.2 (GraphPad Software, Boston, MA, USA). Fisher’s exact test was used to compare categorical variables while Student’s *t*-test or Mann–Whitney *U* test was used to compare continuous variables based on normality (Shapiro–Wilk test). Differences were considered significant at *P*-value <0.05.

## Results

### High-frequency rate of *PLCZ1* and *ACTL7A* variants detected in patients with low or total FF after ICSI

Genetic screening of *PLCZ1, ACTL7A*, and *ACTL9* genes in male patients suffering from FF after ICSI (mean fertilization rate = 14.61%) (Supplementary Table S1) revealed that 29.09% (16/55) of the patients carried at least one variant in *PLCZ1*, 14.81% (4/27) in *ACTL7A*, and 3.70% (1/27) in *ACTL9* (Table 1). Variant classification was adjusted after functional testing performed in this study (Table 1).

**Table 1.** Variants of interest detected in *PLCZ1, ACTL7A*, and *ACTL9* in male patients with total fertilization failure or low fertilization (≤33.33%) after ICSI (P1–P55).

Cohort	P	Gene	Exon	c.DNA change	aa change	**State** (HOM/HET)	RefSNP	**gnomAD** ^a^	**REVEL score** ^b^	**Alpha Missense score** ^c^	**ClinGen Bayesian Classification** (prior to functional testing)^d^	**ClinGen Bayesian Classification** (after functional testing)^d^
**1**	**P2**	PLCZ1	6	c.698A>T	p.His233Leu	HET	rs200061726	0.0009183	0.256	0.171	LP (*P* = 0.988)	P (*P* = 0.994)
		PLCZ1	9	c.964A>T	p.Lys322*	HET	NA	0.00000159	–	–	LP (*P* = 0.9)	LP (*P* = 0.949)
	**P3**	PLCZ1	13	c.1499C>T	p.Ser500Leu	HET	rs10505830	0.04151	0.099	0.086	VUS (hot; *P* = 0.812)	LP (*P* = 0.9)
	**P4**	PLCZ1	13	c.1499C>T	p.Ser500Leu	HET	rs10505830	0.04151	0.099	0.086	VUS (hot; *P* = 0.812)	LP (*P* = 0.9)
	**P7**	PLCZ1	13	c.1499C>T	p.Ser500Leu	HOM	rs10505830	0.04151	0.099	0.086	VUS (hot; *P* = 0.812)	LP (*P* = 0.9)
	**P9**	PLCZ1	5	c.422G>A	p.Arg141His	HET	rs202034240	0.00002366	0.059	0.074	VUS (tepid; *P* = 0.5)	VUS (warm; *P* = 0.675)
	**P15**	PLCZ1	13	c.1499C>T	p.Ser500Leu	HET	rs10505830	0.04151	0.099	0.086	VUS (hot; *P* = 0.812)	LP (*P* = 0.9)
	**P18**	PLCZ1	13	c.1499C>T	p.Ser500Leu	HOM	rs10505830	0.04151	0.099	0.086	VUS (hot; *P* = 0.812)	LP (*P* = 0.9)
	**P20**	PLCZ1	13	c.1499C>T	p.Ser500Leu	HOM	rs10505830	0.04151	0.099	0.086	VUS (hot; *P* = 0.812)	LP (*P* = 0.9)
	**P21**	PLCZ1	6	c.698A>T	p.His233Leu	HET	rs200061726	0.0009183	0.256	0.171	LP (*P* = 0.988)	P (*P* = 0.994)
	**P23**	PLCZ1	13	c.1499C>T	p.Ser500Leu	HOM	rs10505830	0.04151	0.099	0.086	VUS (hot; *P* = 0.812)	LP (*P* = 0.9)
	**P26**	PLCZ1	13	c.1499C>T	p.Ser500Leu	HET	rs10505830	0.04151	0.099	0.086	VUS (hot; *P* = 0.812)	LP (*P* = 0.9)
		PLCZ1	4	c.280C>T	p.Gln94*	HET	rs138801851	0.0001370	–	–	LP (*P* = 0.9)	LP (*P* = 0.949)
**2**	**P29**	ACTL7A	1	c.1088dup	p.Ser364Glnfs*9	HOM	rs752334307	0.00006134	–	–	LP (*P* = 0.949)	P (*P* = 0.997)
	**P32**	PLCZ1	4	c.221T>C	p.Ile74Thr	HET	rs145549980	0.0002654	0.045	0.068	VUS (tepid; *P* = 0.5)	VUS (tepid; *P* = 0.5)
		ACTL7A	1	c.547T>C	p.Tyr183His	HET	rs41278345	0.002240	0.704	0.880	VUS (hot; *P* = 0.812)	VUS (warm, *P* = 0.675)
	**P34**	PLCZ1	6	c.698A>T	p.His233Leu	HET	rs200061726	0.0009183	0.256	0.171	LP (*P* = 0.988)	P (*P* = 0.994)
		ACTL9	1	c.812G>C	p.Arg271Pro	HET	rs73507819	0.02063	0.185	0.287	VUS (cool; *P* = 0.188)	VUS (cool; *P* = 0.188)
	**P35**	PLCZ1	6	c.698A>T	p.His233Leu	HET	rs200061726	0.0009183	0.256	0.171	LP (*P* = 0.988)	P (*P* = 0.994)
	**P36**	PLCZ1	10	c.1136T>C	p.Ile379Thr	HET	rs201548309	0.00005206	0.442	0.533	VUS (hot; *P* = 0.812)	LP (*P* = 0.9)
	**P44**	PLCZ1	6	c.698A>T	p.His233Leu	HET	rs200061726	0.0009183	0.256	0.171	LP (*P* = 0.988)	P (*P* = 0.994)
	**P46**	ACTL7A	1	c.640G>A	p.Gly214Ser	HOM	rs41278347	0.005301	0.891	0.798	VUS (tepid; *P* = 0.5)	VUS (tepid; *P* = 0.5)
		ACTL7A	1	c.1117C>T	p.Arg373Cys	HOM	rs775405375	0.00002540	0.910	0.947	P (*P* = 0.994)	P (*P* = 0.994)
	**P54**	ACTL7A	1	c.1018G>A	p.Val340Met	HET	rs7872077	0.01517	0.197	0.113	VUS (cold; *P* = 0.188)	VUS (cool; *P* = 0.325)
		ACTL7A	1	c.657G>A	p.Val219=	HET	rs3739693	0.01408	**–**	–	VUS (ice cold; *P* = 0.1)	VUS (cold; *P* = 0.188)

a Allele frequency from GnomAD v4.0.0. Interesting variants were filtered when allele frequency < 5%.

b REVEL (rare exome variant ensemble learner) predicts pathogenicity of missense variants based on the prediction of individual tools (MutPred, FATHMM, VEST, Poly-Phen, SIFT, PROVEAN, MutationAssessor, MutationTaster, LRT, GERP, SiPhy, phyloP, and phastCons). A REVEL score above 0.5 indicates pathogenicity.

c AlphaMissense is a machine learning model that predicts the pathogenicity of missense variants based on dual learned protein structure and evolutionary features. AlphaMissense classifies variants with a pathogenicity score as follows: 0–0.34 (likely benign), 0.34–0.564 (uncertain), and 0.564–1 (likely pathogenic).

d Variant classification was performed using the VCT 2020.2 tool from the Center of Medical Genetics Ghent. Final classification is reported according to the ACMG/AMP guidelines converted to a Bayesian framework (Tavtigian *et al.*, 2018) before and after considering functional testing performed in this study. The classification criteria selected for each variant are detailed in Supplementary Table S5.

HET, heterozygous; HOM, homozygous; LP, likely pathogenic; NA, not available; *P*, posterior probability; P, pathogenic; VUS, variant of uncertain significance.

**Figure 1.**
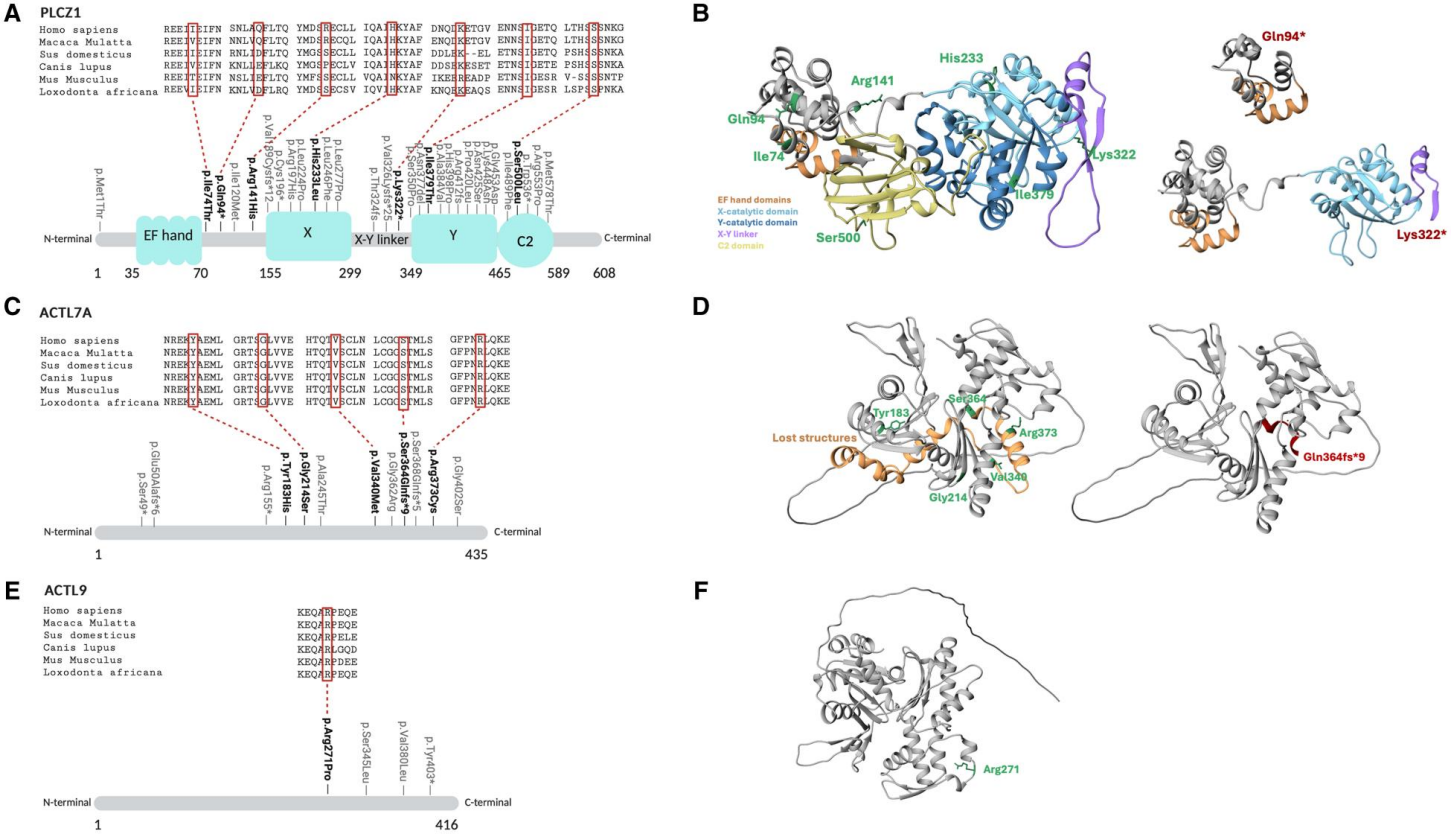
**Variants of interest detected in *PLCZ1, ACTL7A*, and *ACTL9* in male patients with previous total fertilization failure or low fertilization (**≤**33.33%) after ICSI**. Protein location and amino acid conservation of identified *PLCZ1* (**A**), *ACTL7A* (**C**), and *ACTL9* (**E**) variants. Variants identified in this article are in bold type and variants previously reported are in grey type. Affected amino acids are compared across six different mammalian species. In addition, the predicted three-dimensional wild-type PLCZ1 (**B**), ACTL7A (**D**), and ACTL9 (**F**) protein structures, as well as the predicted structural changes of the detected protein-truncating *PLCZ1* (B) and *ACTL7A* (D) variants are depicted. Wild-type amino acid residues are highlighted in green while the protein-truncating variants in red. Predicted three-dimensional protein structures were obtained from AlphaFold. Figures were prepared with USCF Chimera. Effects of missense variants on protein structure can be found in Supplementary Fig. S1.

In total, seven different *PLCZ1* variants were identified affecting distinct PLCζ protein domains (Fig. 1A). Variants in the X and Y domains of PLCζ exhibit high amino acid conservation across species, unlike those in the linker regions (Fig. 1A). All identified variants were novel, except for p.His233Leu and p.Ser500Leu, which have been previously reported in patients with poor fertilization after ICSI (Kashir *et al.*, 2012; Torra-Massana *et al.*, 2019). Interestingly, those two variants were also frequently found in our patient cohort, in 9.09% (5/55) and 14.55% (8/55) of cases, respectively (Table 1). Variant p.His233Leu was identified in a heterozygous state and was classified as pathogenic based on previously reported functional data, which was confirmed in this study. In addition, the brother of P44, who also suffered from FF after ICSI, also carried the *PLCZ1* p.His233Leu variant in a heterozygous state (data not shown). Variant p.Ser500Leu was found both in heterozygous and homozygous states and classified as likely pathogenic after functional testing and given previous data (Yoon *et al.*, 2008; Torra-Massana *et al.*, 2019) (Table 1) although the frequency in gnomAD was relatively high (>0.04151). Two patients were compound heterozygous for variants in the *PLCZ1* gene. P2 exhibited variants p.His233Leu and p.Lys322*, while P26 exhibited variants p.Ser500Leu and p.Gln94* (Table 1). The protein-truncating variants p.Lys322* and p.Gln94* result in the loss of PLCζ catalytic domains (Fig. 1B) and are classified as likely pathogenic. The remaining novel missense variants (p.Ile74Thr, p.Arg141His, and p.Ile379Thr) were also detected in a heterozygous state and classified as VUS, VUS, and likely pathogenic, respectively (Table 1). Strikingly, pathogenesis predictive REVEL and AlphaMissense scores were lower than 0.5 for all missense *PLCZ1* variants, except for p.Ile379Thr, which falls in the uncertain category (Table 1). Moreover, Missense3D did not detect any structural damage in the predicted PLCZ1 three-dimensional protein structure caused by the identified missense variants, at least for the 17 structural features analysed (Supplementary Fig. S1).

Five different variants were found in *ACTL7A,* of which four were novel, and one variant was found in *ACTL9*; all were located in highly conserved sites across different species (Fig. 1C and E). Variant p.Ser364Glnfs*9 (c.1088dup) was detected in a homozygous state in P29 and classified as pathogenic (Table 1). Remarkably, a different *ACTL7A* variant (c.1101dup, p.Ser368Glnfs*5), previously associated with low fertilization after ICSI (Chen *et al.*, 2022), truncates the protein at amino acid position 373, mirroring the truncation seen in variant p.Ser364Glnfs*9 (Fig. 1D). Patient P46 exhibited two *ACTL7A* variants in a homozygous state, p.Arg373Cys and p.Gly214Ser, and both are predicted to cause structural damage and show high REVEL and AlphaMissense scores (Table 1, Supplementary Fig. S1). Variant p.Arg373Cys is classified as pathogenic and was recently found in a patient experiencing total FF after ICSI (Wang *et al.*, 2023). Amino acid position 373 in the ACTL7A protein is a mutational hot spot, also affected by other reported variants: p.Arg373His (Wang *et al.*, 2023) in a patient from a consanguineous family suffering from the same infertility phenotype. The variant p.Gly214Ser is classified as VUS (Table 1) as it shows a slightly higher allele frequency, and it was also detected in a heterozygous state in a different patient from our clinic (not reported in this article) who showed a higher fertilization rate after ICSI (45.83%) but poor embryonic development. Patient P54 carried the missense variant p.Val340Met in a heterozygous state along with an additional heterozygous synonymous variant (p.Val219=), both classified as VUS (Table 1). Variant p.Val340Met shows low pathogenicity REVEL and AlphaMissense scores, but Missense3D predicts structural damage of the protein (Supplementary Fig. S1). Strikingly, digenic variants were observed in a heterozygous state in P32 (*PLCZ1* p.Ile74Thr and *ACTL7A* p.Tyr183His) and in P34 (*PLCZ1* p.His233Leu and *ACTL9* p.Arg271Pro) (Table 1). Variant *ACTL7A* p.Tyr183His was classified as VUS as functional testing performed in this study did not corroborate the pathogenicity indicated by the *in silico* tools. Lastly, variant *ACTL9* p.Arg271Pro is classified as VUS, as both REVEL and AlphaMissense pathogenicity scores are low, and no structural damage is detected by Missense3D (Supplementary Fig. S1).

### Calcium pattern analysis revealed low or absent calcium release in mouse and human oocytes during fertilization in patients with *PLCZ1* and *ACTL7A* variants

The MOAT revealed reduced sperm activation potential in mouse oocytes in 11 out of 16 patients with *PLCZ1* variants (activation rates ranging from 20% to 84%) and in three out of four patients with *ACTL7A* variants (activation rates lower than 26%) (Table 2).

**Table 2.** MOAT, MOCA, and HOCA results in male patients with identified *PLCZ1, ACTL7A*, and *ACTL9* variants with previous total fertilization failure or low fertilization (≤33.33%) after ICSI.

			MOAT		MOCA		HOCA	
Cohort	Patient	**Identified variant** (gene, aa change, zygosity)	**Activation rate** (2cell/MII)	Group	N	**Mean AxF** (AU)	N	**Mean AxF** (AU)
1	**P2**	PLCZ1, p.His233Leu, Het PLCZ1, p.Lys322*, Het	19.23% (5/26)	1	17	2.15**	10	0.00**
	**P3**	PLCZ1, p.Ser500Leu, Het	40.00% (8/20)	2	NA	NA	NA	NA
	**P4**	PLCZ1, p.Ser500Leu, Het	77.78% (21/27)	2	NA	NA	NA	NA
	**P7**	PLCZ1, p.Ser500Leu, Hom	83.87% (26/31)	2	15	2.03**	7	0.00**
	**P9**	PLCZ1, p.Arg141His, Het	83.33% (20/24)	2	16	10.35**	8	0.10**
	**P15**	PLCZ1, p.Ser500Leu, Het	50.00% (7/14)	2	NA	NA	NA	NA
	**P18**	PLCZ1, p.Ser500Leu, Hom	54.17% (13/24)	2	11	39.17*	14	0.60**
	**P20**	PLCZ1, p.Ser500Leu, Hom	63.64% (14/22)	2	11	12.40	10	0.00**
	**P21**	PLCZ1, p.His233Leu, Het	89.47% (17/19)	3	10	13.00	13	0.00**
	**P23**	PLCZ1, p.Ser500Leu, Hom	78.57% (22/28)	2	11	18.93**	9	0.00**
	**P26**	PLCZ1, p.Gln94*, Het PLCZ1, p.Ser500Leu, Het	89.66% (26/29)	3	7	33.24*	8	0.00**
2	**P29**	ACTL7A, p.Ser364Glnfs*9, Hom	25.81% (8/31)	1–2	18	0.15**	10	0.00**
	**P32**	ACTL7A, p.Tyr183His, Het PLCZ1, p.Ile74Thr, Het	92.86% (26/28)	3	14	94.63	11	3.24
	**P34**	ACTL9, p.Arg271Pro, Het PLCZ1, p.His233Leu, Het	87.50% (28/32)	3	16	15.53**	13	0.00**
	**P35**	PLCZ1, p.His233Leu, Het	79.17% (19/24)	2	16	22.02**	9	0.00**
	**P36**	PLCZ1, p.Ile379Thr, Het	100.00% (30/30)	3	10	18.17**	10	0.00**
	**P44**	PLCZ1, p.His233Leu, Het	80.00% (20/25)	2	NA	NA	NA	NA
	**P46**	ACTL7A, p.Gly214Ser, Hom ACTL7A, p.Arg373Cys, Hom	25.81% (8/31)	1–2	17	0.53**	10	0.00**
	**P54**	ACTL7A, p.Val340Met, Het ACTL7A, p.Val219=, Het	7.14% (3/42)	1	14	1.08**	11	0.00**
	**C**	–	93.55% (464/496)		42	85.71	58	3.88

Patient results were compared to the control group. *T*-test or Mann–Whitney *U* test to compare MOCA and HOCA outcomes when appropriate. Statistical significance was considered when *P*-value < 0.05.

* *P* < 0.05.

** *P*-value < 0.0001.

A, amplitude; AU, arbitrary units; C, control; F, frequency; HOCA, human oocyte calcium analysis; MOAT, mouse oocyte activation test; MOCA, mouse oocyte calcium analysis; MII, metaphase II oocyte; P, patient.

**Figure 2.**
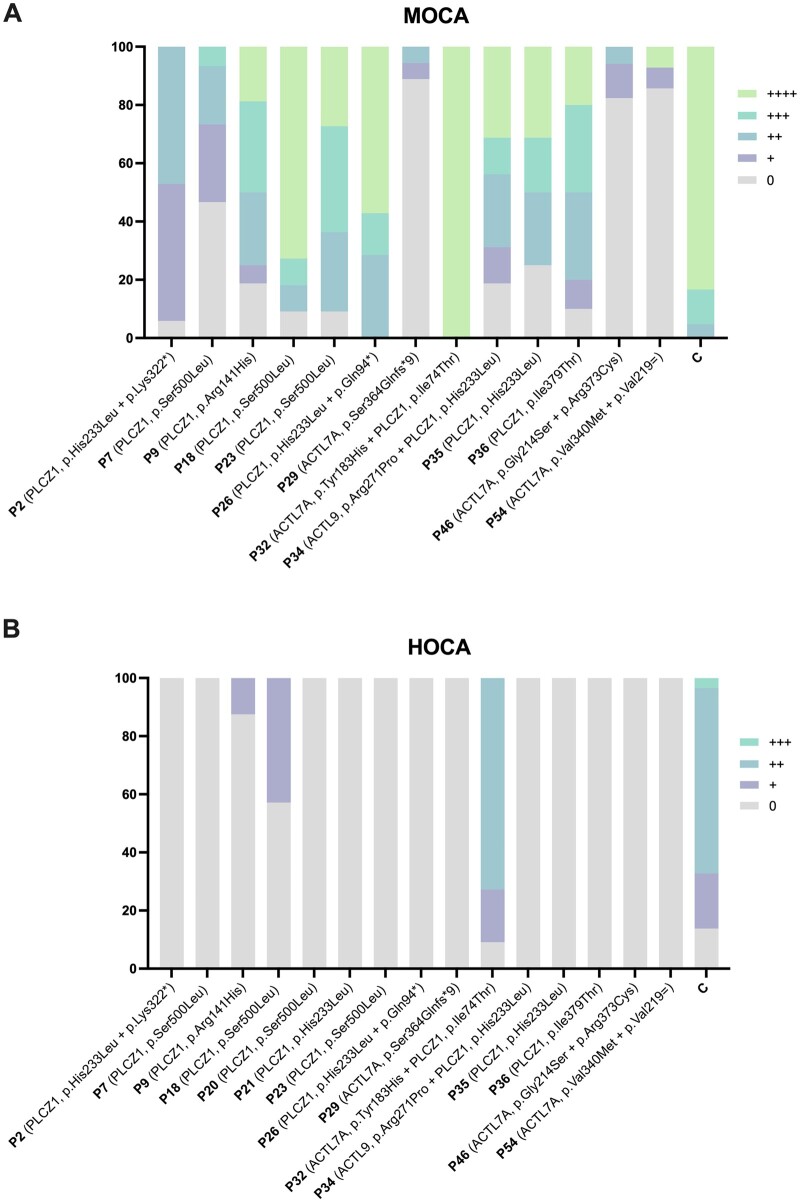
**Frequency scoring of Ca^2+^ oscillations after mouse oocyte calcium analysis (A) and human oocyte calcium analysis (B) in patients with total fertilization failure or low fertilization (**≤**33.33%) after ICSI**. Oocytes were classified according to the number of calcium (Ca^2+^) rises observed during 2 h measurement for MOCA and 10 h measurement for HOCA: ++++: >20 spikes, +++: 10–20 spikes; ++: 3–9 spikes; +: 1–2 spikes; 0: absence of spikes. Representative graphs of the different Ca^2+^ rises observed can be found in Supplementary Fig. S3. MOCA, mouse oocyte calcium analysis; HOCA, human oocyte calcium analysis; P, patient; C, control.

To further determine the effect of *PLCZ1, ACTL7A*, and *ACTL9* variants on the Ca^2+^-releasing capacity of patient sperm cells, and indirectly on the PLCZ1 protein content (Cardona Barberán *et al.*, 2022), the MOCA and HOCA tests were performed. We could not execute any of these tests for P3, P4, P15, and P44, due to the unavailability of sperm samples. The MOCA and HOCA tests showed significantly reduced Ca^2+^ release in comparison to a fertile control sample in all patients analysed, except for P32 (carrier of variants *PLCZ1* p.Ile74Thr and *ACTL7A* p.Tyr183His) (Table 2), which was largely above the threshold for normal fertilization corresponding to A×F >9 and >0.6 in the MOCA and HOCA tests, respectively (Vanden Meerschaut *et al.*, 2013; Ferrer-Buitrago *et al.*, 2018). As such, this indicates that the specific variants found in this patient are unlikely to be the cause of low fertilization after ICSI. Patients with homozygous *ACTL7A* variants (P29 and P46) and P54, with compound heterozygous variants in *ACTL7A*, showed MOCA and HOCA scores below the fertilization threshold (Table 2). However, an A×F score >9 was observed during MOCA for most patients with *PLCZ1* detected variants, although HOCA revealed the absence of or very low Ca^2+^release with A×F score ≤0.6 (Table 2), including patients with heterozygous *PLCZ1* variants. In addition, when assessing the frequency of Ca^2+^ spikes induced per patient into mouse oocytes (Fig. 2A, Supplementary Fig. S3A), a high heterogeneity was observed between sperm carriers of different variants (*PLCZ1* vs *ACTL7A*), but also between sperm carriers of the same variant (e.g. P7, P18, and P23 carriers of *PLCZ1* homozygous p.Ser500Leu variant) (Fig. 2A). Only a few sperm cells from P9 and P18 were able to trigger one to two Ca^2+^ peaks in human oocytes, while most sperm cells harbouring identified variants (excluding P32), were unable to elicit Ca^2+^ oscillations in human oocytes (Fig. 2B, Supplementary Fig. S3B).

### Deleterious effects of *ACTL7A* variants on protein expression and acrosome structure

To assess the impact of *ACTL7A* variants on protein location and expression, immunofluorescence staining was conducted in patient sperm samples. In addition, to confirm whether the identified *ACTL7A* and *ACTL9* variants alter acrosome structure, sperm head morphology was evaluated by transmission electron microscopy (TEM). Frozen–thawed samples were used for this analysis. Due to the impact of the cryopreservation process on acrosome integrity (Sun *et al.*, 2020) and the inherent acrosome morphological variability in sperm samples (Skowronek *et al.*, 2010), only sperm cells with intact acrosomes were included in these analyses (Supplementary Fig. S4 and Supplementary Table S6).

**Figure 3.**
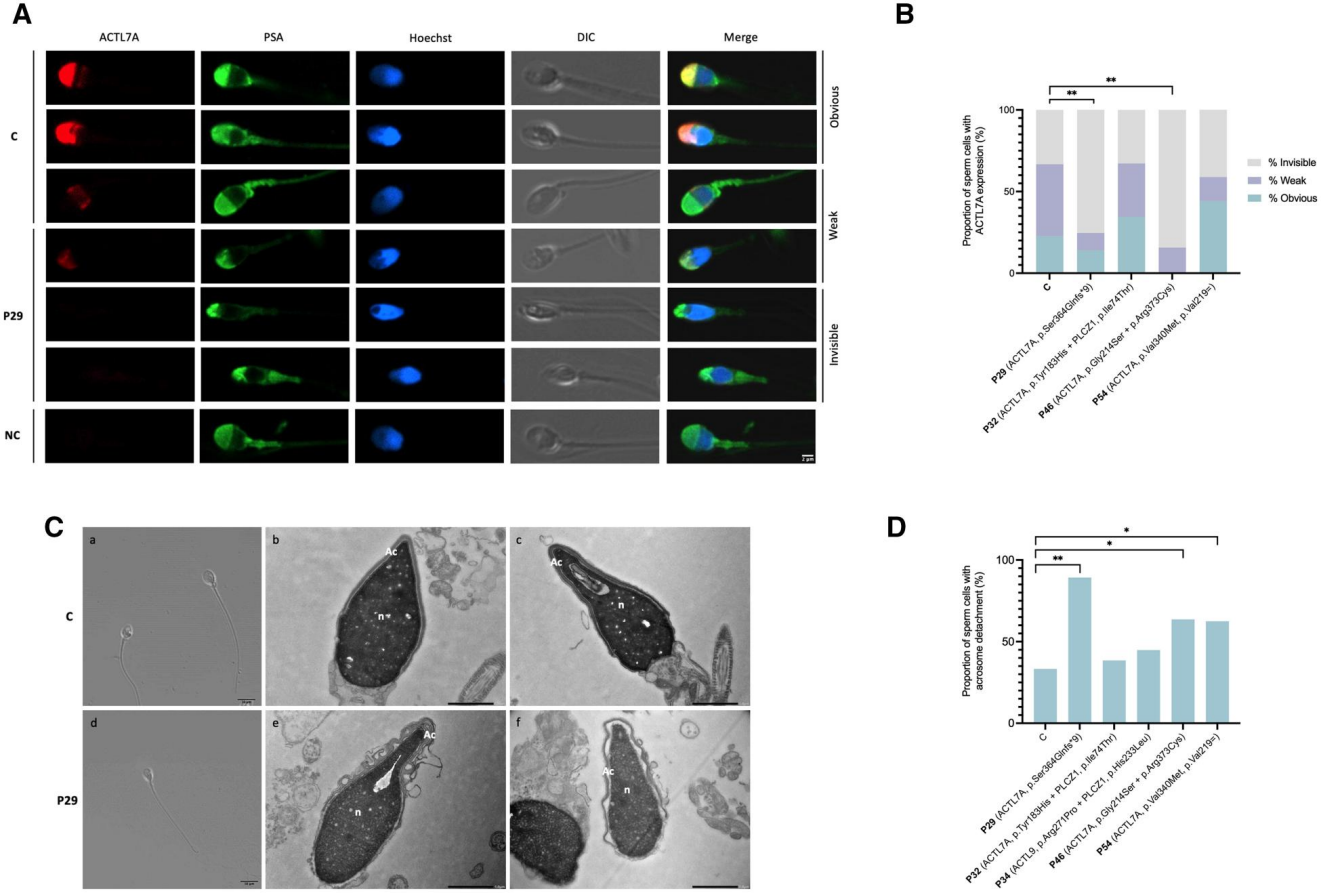
**Functional analysis of *ACTL7A* and *ACTL9* identified variants**. (**A**) Representative images of ACTL7A immunofluorescence of sperm cells from a fertile control and P29. Localization of ACTL7A protein is detected in red, the acrosome is labelled with PSA-FITC (green) and DNA with Hoechst (blue). ACTL7A expression (obvious and weak signal) was observed in the sperm head of control samples, while in P29, most sperm cells showed no ACTL7A expression (invisible signal). Scale bar = 2 μm. (**B**) Proportion of sperm cells with invisible, weak, or obvious ACTL7A expression in a fertile control and patients with *ACTL7A* variants. (**C**) Representative images of a fertile control and P29 sperm head morphology using optical microscopy at 100× (a and d) and transmission electron microscopy (TEM) at 10 000× (b, c, e, and f). Scale bar = 10 μm (a and d). Scale bar = 1 μm (b, c, e, and f). Normal acrosome morphology is observed in control sperm (b and c), while the acrosome appears folded, curved, and detached from the nuclear envelope in P29 (e and f). (**D**) Acrosome detachment rate observed after transmission electron microscopy (TEM) analysis of sperm head ultrastructure in patients with *ACTL7A* and *ACTL9* variants in comparison to a control sample. Rates were compared using Fisher’s exact test. *P < 0.05, **P-value < 0.0001. C, control; P, patient; DIC, differential interference contrast; NC, negative control; Ac, acrosome; n, nucleus.

*ACTL7A* immunofluorescence staining in control samples revealed that ACTL7A colocalized with the acrosomal marker PSA (Fig. 3A). A total of 66.67% control sperm cells showed ACTL7A expression (Fig. 3B). In contrast, ACTL7A signal in patients with homozygous *ACTL7A* variants (P29 and P46) appeared unevenly distributed or was invisible for most sperm cells analysed (Fig. 3A), with a significantly reduced percentage of sperm cells showing ACTL7A expression (24.56% and 15.69%, respectively) (Fig. 3B). However, P32 and P54 containing heterozygous variants had a similar proportion of sperm cells with ACTL7A expression (67.19% and 58.82%, respectively) (Fig. 3B).

TEM analysis revealed a significantly increased acrosome detachment rate for some patients with *ACTL7A* variants in comparison to the control sample (33.33%) (Fig. 3C and D). The highest acrosome detachment rate (89.29%) was observed in P29, a homozygous carrier of a frameshift variant. Sperm heads from P29 under optical microscopy appeared to be normal, but TEM revealed acrosome detachment from the nuclear envelope, and in most sperm cells, the acrosome showed a folded and curved shape (Fig. 3C). In addition, P46, a homozygous carrier of missense variants in *ACTL7A*, showed 63.64% acrosome detachment, while P54, a compound heterozygous carrier of ACTL7A variants, showed 62.50% acrosome detachment. The other patients analysed, P32 and P34 who presented digenic variants in *PLCZ1* and in *ACTL7A* or *ACTL9*, respectively, did not show an increased rate of acrosome detachment (38.46% and 44.83%) (Fig. 3D).

### ICSI–AOA treatment using ionomycin restores fertilization and pregnancy rates in patients with *PLCZ1* and *ACTL7A* variants

**Figure 4.**
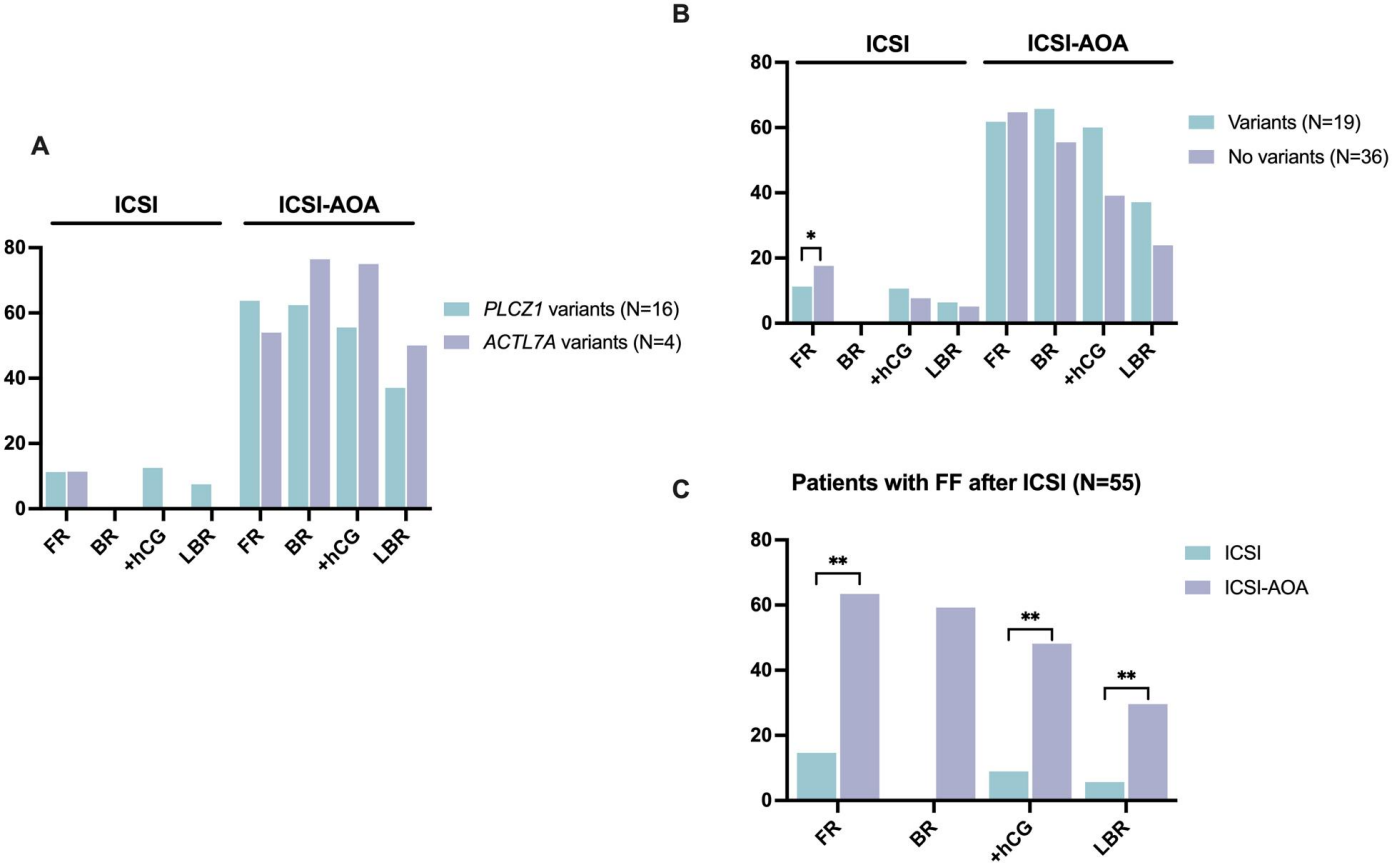
**Comparison of clinical outcomes after ICSI and ICSI–AOA cycles in male patients with total fertilization failure or low fertilization (**≤**33.33%) after ICSI**. Comparison of fertilization rate (FR), blastocyst rate (BR), positive hCG rate (+hCG), and live birth rate (LBR) after ICSI and ICSI–assisted oocyte activation (AOA) treatments: (**A**) between patients with *PLCZ1* and *ACTL7A* variants; (**B**) between patients with and without detected variants in *PLCZ1, ACTL7A*, and *ACTL9* genes; and (**C**) in all patients with low or total fertilization failure after ICSI included in the study. Rates were compared using Fisher’s exact test. *P < 0.05, **P-value < 0.0001.

ICSI–AOA treatment with ionomycin led to a significant increase in fertilization rates among the 19 patients carrying *PLCZ1, ACTL7A*, and *ACLT9* variants. Fertilization rates improved from 11.24% in 47 conventional ICSI cycles to 61.80% in 35 ICSI–AOA cycles (*P* < 0.0001) (Table 3). In addition, a high blastocyst rate of 65.73% was obtained in the AOA cycles where D5 embryo transfer was performed. In total, after ICSI–AOA cycles, 21 embryo transfers were performed on D2 or D3 while 37 were transferred on D5. Positive hCG rate per cycle increased from 10.64% after ICSI to 60.00% after ICSI–AOA (*P* < 0.0001), while live birth rate per cycle increased from 6.38% after ICSI to 37.14% after ICSI–AOA (*P* = 0.0007), resulting in 13 healthy children (Table 3). AOA treatment clearly benefitted these patients as only three live births were achieved in the former ICSI cycles from a total of 24 embryo transfers on D2 or D3 and 3 on D5. Notably, no differences were observed in the ICSI–AOA outcome between patients with *PLCZ1* variants and *ACTL7A* variants (Fig. 4A), although the blastocyst rate was slightly higher for patients with *ACTL7A* variants. Importantly, all four patients with *ACTL7A* variants successfully achieved pregnancy (Table 3). Patients P29 and P32 had two healthy live births each, while P46 and P54 have an ongoing pregnancy (Supplementary Table S4).

**Table 3.** Clinical outcomes after ICSI–AOA cycles in male patients with identified variants who suffered from previous total fertilization failure or low fertilization (≤33.33%) after ICSI.

			ICSI cycles				ICSI–AOA cycles				
Cohort	Patient	**Identified variant**(gene, aa change, zygosity)	N	**Fertilization rate** (2PN/MII)	**N embryos transferred** (Day of transfer)	N positive hCG and clinical outcome per cycle	N	**Fertilization rate after** (2PN/MII)	**Blastocyst rate** (blastocyst/2PN)	**N embryos transferred** (Day of transfer)	N positive hCG and clinical outcome per cycle
**1**	**P2**	PLCZ1, p.His233Leu, Het PLCZ1, p.Lys322*, Het	5	7.69% (5/65)	1 (D2) + 2 (D3)	1, LB	1	40.00% (8/20)	NA	5 (D3)	1, LB
	**P3**	PLCZ1, p.Ser500Leu, Het	1	0.00% (0/10)	–	–	8	68.25% (43/63)	NA	7 (D3) + 8 (D2)	1, ND
	**P4**	PLCZ1, p.Ser500Leu, Het	2	10.00% (1/10)	0	–	1	83.33% (5/6)	NA	1 (D3)	0
	**P7**	PLCZ1, p.Ser500Leu, Hom	4	2.47% (2/81)	1 (D2)	0	1	80.00% (12/15)	50.00% (6)	2	1, LB
	**P9**	PLCZ1, p.Arg141His, Het	3^a^	0.00% (0/18)	–	–	2	30.77% (4/13)	0.00% (0)	–	–
	**P15**	PLCZ1, p.Ser500Leu, Het	3	31.25% (5/16)	5 (D3)	0	3	28.57% (2/7)	100.00% (2)	1	1, BP
	**P18**	PLCZ1, p.Ser500Leu, Hom	5	25.81% (16/62)	3 (D3)	1, M	1	61.54% (8/13)	62.50% (5)	2	2, BP, LB
	**P20**	PLCZ1, p.Ser500Leu, Hom	2	18.75% (3/16)	2 (D3)	0	1	61.54% (8/13)	87.50% (7)	5	2, 2xLB
	**P21**	PLCZ1, p.His233Leu, Het	1	0.00% (0/8)	–	–	1	76.47% (13/17)	38.46% (5)	1	1, LB
	**P23**	PLCZ1, p.Ser500Leu, Hom	4	11.54% (3/26)	2 (D3)	1, LB	2	62.50% (10/16)	80.00% (8)	3	1, M
	**P26**	PLCZ1, p.Gln94*, Het PLCZ1, p.Ser500Leu, Het	1	0.00% (0/11)	–	–	1	77.78% (14/18)	71.43% (10)	5	1, BP
**2**	**P29**	ACTL7A, p.Ser364Glnfs*9, Hom	1	13.33% (2/15)	1 (D3)	0	1	81.82% (9/11)	66.67 (6)	3	2, 2xLB
	**P32**	ACTL7A, p.Tyr183His, Het PLCZ1, p.Ile74Thr, Het	1	25.00% (1/4)	0	–	5^a^	52.00% (13/25)	84.61% (11)	6^c^	2, 2xLB
	**P34**	ACTL9, p.Arg271Pro, Het PLCZ1, p.His233Leu, Het	2	0.00% (0/25)	–	–	1	100.00% (11/11)	72.73% (8/11)	2	2, 1xLB, 1xBP
	**P35**	PLCZ1, p.His233Leu, Het	2	6.67% (1/15)	0	–	2	45.16% (14/31)	50.00% (7)	2	1, LB
	**P36**	PLCZ1, p.Ile379Thr, Het	2	19.05% (4/21)	4 (D3)	1, M	1	100.00% (7/7)	71.43% (5)	1^c^	0
	**P44**	PLCZ1, p.His233Leu, Het	3	20.00% (7/35)	1 (D3) + 2 (D5)	1, LB	1	66.67% (6/9)	83.33% (5)	1	1, LB
	**P46**	ACTL7A, p.Gly214Ser, Hom ACTL7A, p.Arg373Cys, Hom	3	8.70% (4/46)	1 (D3)	0	1	26.32% (5/19)	60.00% (3)	2	1, Ong
	**P54**	ACTL7A, p.Val340Met, Het ACTL7A, p.Val219=, Het	2^b^	14.29% (2/14)	1 (D5)	0	1	87.50% (7/8)	85.71% (6)	1	1, Ong
	**TOTAL**		47	11.24% (56/498)	27	+hCG = 10.64% (5/47) LBR = 6.38% (3/47)	35	61.80%** (199/322)	65.73% (94/143)^d^	58	+hCG = 60.00%** (21/35) LBR = 37.14%* (13/35)

a Oocyte donation was used in the last ICSI cycle from P9 and the last ICSI–AOA cycles from P32. One live birth from P32 was achieved with oocyte donation.

b P54 did not undergo an ICSI cycle but was treated directly with ICSI–AOA using calcium ionophore. The specific AOA protocol used is not known.

c PGT was needed in these couples, thus blastocysts that carried the corresponding mutation were not used.

d Blastocyst rate is calculated over the fertilized oocytes from cycles with blastocyst culture. Total fertilization and pregnancy rates between ICSI and ICSI–AOA cycles were compared using Fisher’s exact test,

* *P*-value < 0.005 and

** *P*-value < 0.0001.

BP, biochemical pregnancy; D, day; Het, heterozygous; Hom, homozygous; LB, live birth; LBR, live birth rate; M, miscarriage; MII, metaphase II oocyte; ND, neonatal death; Ong, ongoing pregnancy; 2PN, two pronuclei; +hCG, positive hCG rate.

ICSI–AOA treatment with ionomycin has been repetitively reported as a very effective treatment for couples with FF after ICSI (Bonte *et al.*, 2019). When comparing ICSI–AOA efficiency in patients with and without variants (Fig. 4B), similar fertilization rates were achieved (61.80% and 64.72%, respectively). Although no significant difference was detected in the blastocyst, positive hCG and live birth rates between both groups, considerably lower rates were obtained in the group with no detected variants (55.47%, 39.13% and 23.91.%, respectively) in comparison to couples with detected variants (65.73%, 60.00%, and 37.14%, respectively) (Fig. 4B).

Overall, for the 55 patients included in this study (with and without variants), ICSI–AOA treatment significantly increased the fertilization rate, as well as positive hCG and live birth rates per cycle (Fig. 4C). In total, 25 births were achieved after ICSI–AOA in which no major congenital malformations were observed, except one child who was born with only one normal functioning kidney, the cause of which was unknown (Supplementary Table S7). One birth ended up in neonatal death. Sex distribution, mean birth weight, and Apgar scores were all normal. In addition, four pregnancies are still ongoing (Supplementary Table S4).

## Discussion

To our knowledge, this is the first study to perform targeted-genetic screening of the male genes *PLCZ1, ACTL7A*, and *ACTL9* in a large cohort of Caucasian patients with total FF or low fertilization (≤33.33%) rates (FF) after ICSI. We expanded the spectrum of known variants, revealing high detection rates, with *PLCZ1* variants identified in 29.09% of our patients, consistent with previous findings (Torra-Massana *et al.*, 2019; Dai *et al.*, 2020; Yan *et al.*, 2020). *ACTL7A* variants were found in 14.81% of cases, while *ACTL9* variants were less prevalent (3.70%). Previous studies have reported *ACTL7A* and *ACTL9* variant detection rates of 8.33% (Chen *et al.*, 2022) and 14.29% (Dai *et al.*, 2021), in 12 and 21 patients with low fertilization rates (<20%) following ICSI, respectively. Most publications typically describe a few isolated cases from consanguineous families, particularly within the Chinese population (Wang *et al.*, 2023; Zhao *et al.*, 2023).

From the seven identified *PLCZ1* variants, those affecting the X (p.His233Leu) and Y catalytic (p.Ile379Thr) domains, or those truncating the protein before these domains (p.Gln94* and p.Lys233*), exhibit the highest impact on protein function. These domains are crucial for generating inositol 1,4,5-triphosphate (IP3), which induces Ca^2+^ release from the endoplasmic reticulum stores to the oocyte cytoplasm (Thanassoulas *et al.*, 2022). Injecting *PLCZ1* p.His233Leu complementary RNA (cRNA) into a mouse (Kashir *et al.*, 2012) and human oocytes (Torra-Massana *et al.*, 2019) resulted in abnormal Ca^2+^ oscillations and fewer fertilized oocytes compared to wild-type. In our study, all patients with this variant (n = 5) showed reduced Ca^2+^ release during MOCA and no release during HOCA. The novel variants p.Gln94*, p.Lys233*, p.Ile379Thr, and p.Arg141His also failed to release Ca^2+^ ions in human oocytes. In contrast, the p.Ile74Thr showed normal Ca^2+^ release suggesting no pathogenicity. The p.Ser500Leu variant, located in the C2 domain which is crucial for substrate binding to PI_3_P and PI_5_P membrane phospholipids (Thanassoulas *et al.*, 2022), was deemed likely pathogenic following our functional testing. Despite its relatively high frequency in gnomAD, it is more prevalent in the study population (14.55%). MOCA and HOCA showed reduced or absent Ca^2+^ release in mouse and human oocytes in all patients with homozygous and even heterozygous p.Ser500Leu *PLCZ1* variants. However, p.Ser500Leu *PLCZ1* cRNA injection into a limited number of human oocytes resulted in normal fertilization rates (Torra-Massana *et al.*, 2019). Discrepancies in these tests could have been the result of injecting a cRNA concentration exceeding physiological levels and to the difficulty in maintaining uniform cRNA injection volume (Yamaguchi *et al.*, 2017). Nonetheless, when evaluating the results of the mouse tests (MOAT and MOCA), it is crucial to consider interspecies differences, as the human PLCζ protein is more potent than the mouse version (Nomikos *et al.*, 2014). This disparity may explain why some patients with variants display higher activation rates in mouse oocytes (A×F > 9 scores) (Vanden Meerschaut *et al.*, 2013) but experience absent Ca^2+^ release in human oocytes (A×F ≤ 0.6). Future studies should reconsider the threshold for distinguishing between low and normal fertilization rates after ICSI considering patients with *PLCZ1* variants, currently set at A×F ≤ 9 for MOCA and A×F ≤ 0.6 for HOCA (Ferrer-Buitrago *et al.*, 2018). At present, HOCA remains the most sensitive test for identifying pathogenic variants, albeit requiring human oocytes.

In this study, we also report new pathogenic *ACTL7A* variants causing FF after ICSI and confirm their impact on acrosome ultrastructure and PLCζ protein levels through Ca^2+^ imaging. Two different *Actl7a^−^*^/^^*−*^ mouse models support our findings (Xin *et al.*, 2020; Zhou *et al.*, 2022), showing that an abnormal Actl7a protein in mice sperm causes infertility, compromised acrosome morphology, reduced PLCζ protein content, and impaired Ca^2+^ release, leading to total FF after both ICSI and IVF (Xin *et al.*, 2020; Zhou *et al.*, 2022). Similarly, in our study, sperm with homozygous *ACTL7A* variants (p.Ser364Glnfs*9 and p.Arg373Cys) failed to induce Ca^2+^ release in human oocytes, consistent with previous findings of decreased or absent PLCζ fluorescence intensity in patients with *ACTL7A* variants (Wang *et al.*, 2021; Chen *et al.*, 2022; Dai *et al.*, 2022a; Zhou *et al.*, 2023). Both variants also caused acrosome detachment from the nuclear envelope and reduced ACTL7A fluorescence intensity in patients’ sperm. P46, carrying the p.Arg373Cys *ACTL7A* variant, also showed a VUS homozygous variant p.Gly214Ser in *ACTL7A*, the causality of which remains unclear from our functional tests, but is predicted to be less pathogenic than p.Arg373Cys (Wang *et al.*, 2023). Patient P54, with compound heterozygous variants in *ACTL7A* (p.Val340Met and p.Val219=) categorized as VUS, also showed low activation rates after MOAT, reduced Ca^2+^ release, and increased acrosome detachment, despite normal ACTL7A protein levels. Further investigation is needed to assess the impact of p.Val340Met on the ACTL7A protein structure or whether other male factors contribute to this phenotype. In contrast, P32 containing heterozygous *ACTL7A* p.Tyr183His and *PLCZ1* p.Ile74Thr variants showed no Ca^2+^ release deficiency or acrosome abnormalities, and normal ACTL7A fluorescence intensity. ICSI–AOA increased the fertilization rate from 25% (1/4) after ICSI to 45% (9/20) in four cycles, which is suboptimal compared to the typical 70% recovery in confirmed male factor cases (Bonte *et al.*, 2019). Nonetheless, the blastocyst rate increased to 66% and a live birth was obtained. This couple also underwent one ICSI–AOA cycle with oocyte donation (Supplementary Table S4) and in this case fertilization rate improved to 80% (4/5) resulting in another live birth. These data point more towards a female factor. However, we cannot completely exclude the possibility of a combined factor, with defects in other male undiscovered proteins affecting fertilization. Lastly, P34, carrying heterozygous *ACTL9* p.Arg271Pro and *PLCZ1* p.His233Leu variants, showed normal acrosome formation but abnormal Ca^2+^ release. The *ACTL9* p.Arg271Pro variant, classified as VUS with benign criteria, is unlikely to be responsible for the total FF observed after ICSI. The causative variant appears to be exclusively *PLCZ1* p.His233Leu, known to cause FF after ICSI even in heterozygous state.

Based on the combined TEM and immunofluorescence data, only homozygous *ACTL7A* variants cause decreased ACTL7A protein levels and consequently acrosome detachment. This aligns with the ACTL7A protein’s role, which participates in the formation and fusion of Golgi-derived vesicles during acrosome biogenesis that starts in the primary spermatocytes (diploid cells) (Xin *et al.*, 2020).

A novel aspect of this article is the description of heterozygous *PLCZ1* variants causing failed fertilization, until now only reported by one group (Torra-Massana *et al.*,2019) while most *PLCZ1* variants in the literature are typically identified in a homozygous or compound heterozygous state (Mu *et al.*, 2020; Yan *et al.*, 2020). Our findings demonstrate that heterozygous *PLCZ1* variants alone can disrupt Ca^2+^ release in all sperm cells from the affected patients. This indicates haploinsufficiency, where haploid sperm cells contain reduced PLCζ protein levels regardless of the presence of the wild-type or a mutant allele. Since mature sperm cells are transcriptionally and translationally inactive (Grunewald *et al.*, 2005; Kleene, 2013) but are functionally equivalent, haploid spermatid cells share mRNAs and proteins via cytoplasmic bridges to minimize phenotypic variability (Bhutani *et al.*, 2021). Although the extent of PLCζ protein sharing remains unclear, it is established that a minimum level of PLCζ protein is required for successful fertilization (Rogers *et al.*, 2004; Yamaguchi *et al.*, 2017). This variability in wild-type PLCZ1 protein among sperm from heterozygous patients, as observed in our MOCA test and in previous immunofluorescence and immunoblotting PLCZ1 studies (Torra-Massana *et al.*, 2019; Peng *et al.*, 2023), explains the diverse fertilization rates and occasional total FF after ICSI detected in some cases. While most of these patients seek ART, intriguingly, some males with heterozygous *PLCZ1* variants can conceive naturally (Dai *et al.*, 2020; Mu *et al.*, 2020). This is in line with the results obtained from the *Plcz1* knock-out mouse model, which shows FF after ICSI but is able to produce offspring after *in vivo* mating, yet at lower rates than wild-type mice (Hachem *et al.*, 2017). These mice also show better fertilization rates after IVF, but with high rates of polyspermy. For individuals with heterozygous *PLCZ1* variants and adequate sperm quality, IVF treatment may be a viable option. It is possible that different oocyte activation mechanisms, for instance, other PLC isoforms, might take over when PLCζ protein content is abnormal during sperm–oocyte fusion, a process bypassed during ICSI. It remains possible that there are other *PLCZ1* variants present in regulatory intronic regions, or in other male genes. For instance, a new male gene, *IQCN*, was recently discovered to cause FF after ICSI (Dai *et al.*, 2022b), also impacting sperm acrosome morphology and reducing PLCζ protein content (Sha *et al.*, 2023).

Finally, AOA treatment with CaCl_2_ injection and ionomycin proved to be very efficient for patients with *ACTL7A* variants, increasing fertilization rates from 11.39% after ICSI to 53.97%, and resulting in four live births and two ongoing pregnancies. Previously, two live births were obtained using in-house prepared calcimycin (A23187) for these patients (Wang *et al.*, 2021; Dai *et al.*, 2022a; Zhao *et al.*, 2023). Our AOA protocol has been consistently reported to be highly effective for FF after ICSI, especially in those cases with confirmed Ca^2+^-releasing deficiencies (Heindryckx *et al.*, 2008; Vanden Meerschaut *et al.*, 2013; Ferrer-Buitrago *et al.*, 2018; Bonte *et al.*, 2019). Here, we report an increase in fertilization rates from 11.22% after ICSI to 63.71% in patients with *PLCZ1* variants, comparable to other reports using in-house prepared ionomycin (Torra-Massana *et al.*, 2019; Yan *et al.*, 2020), but superior to those using in-house prepared calcimycin (Zhao *et al.*, 2023). Notably, our AOA protocol also improved successful outcomes in patients with prior failed fertilization after ICSI–AOA in external centres, particularly those using ready-to-use calcimycin (Supplementary Tables S2 and S4). We have shown the importance of ionophore selection for AOA, as in-house prepared ionomycin gives higher Ca^2+^ release and activation potential than ready-to-use calcimycin (Nikiforaki *et al.*, 2016; Cardona Barberán *et al.*, 2022). AOA treatment is now an evidence-based ART for patients with FF after ICSI (Lundin *et al*., 2023). Studies on the health of children born after AOA show no major congenital defects (Deemeh *et al.*, 2015; Mateizel *et al.*, 2018), as well as no impairments in cognition, motor, and language abilities (Vanden Meerschaut *et al.*, 2014). Nonetheless, long-term follow-up of these children is essential, and caution is advised when considering AOA for other infertility conditions such as embryo developmental arrest (Cardona Barberán *et al.*, 2023) or advanced maternal age and POI (Lv *et al.*, 2020), for which efficacy is still unsure.

Overall, targeted genetic screening of *PLCZ1* and *ACTL7A* simplifies the diagnosis of FF after ICSI, identifying up to one-third of male-related causes and eliminating the need for complex tests like MOAT or Ca^2+^ analysis. For VUS variants, the HOCA test can be used to determine pathogenicity. Reporting *PLCZ1, ACTL7A*, and *ACTL9* variants not only informs about the risk of transmitting subfertility to the offspring but also guides AOA success predictions. Patients with male variants often achieve higher pregnancy rates compared to those without, where female-related genes (e.g. *WEE2, CDC20, TLE6*) may contribute to low fertilization (Cardona Barberán *et al.*, 2022; Wei *et al.*, 2023). Whole-exome sequencing of both genders could uncover new genes causing failed fertilization, particularly in ICSI–AOA failure, and identify genes unrelated to Ca^2+^ dynamics. This may lead to the development of new therapies, such as wild-type cRNA injection (Sang *et al.*, 2018) or cytoplasm donation (spindle transfer) which have been suggested for specific female-related FF (Tang *et al.*, 2022; Costa-Borges *et al.*, 2023).

## Supplementary Material

hoae057_Supplementary_Data

## Data Availability

The data underlying this article are available in the article and in its online supplementary material.
