# Supplementary material for: High rate of detected variants in male PLCZ1 and ACTL7A genes causing failed fertilization after ICSI
Source: Hum Reprod Open. 2024 Sep 28;2024(4):hoae057. doi: 10.1093/hropen/hoae057 (PMC11479693; doi:10.1093/hropen/hoae057)
Supplement: hoae057_Supplementary_Data [file hoae057_supplementary_data.zip › HRO-24-0042-R2-SuppTables1-7.docx.docx]

**Supplementary Table S1 Clinical outcomes after ICSI cycles in patients with total fertilization failure or low fertilization (**≤**33.33%) (P1-P55).**

| **Cohort** | **Patient** | **Age**  (in the last ICSI cycle) | **ICSI cycles, n** | **External** (E)/ **Internal** (I) **cycles^a^** | **Fertilization rate** (2PN/MII) | **N embryos transferred**  (Day of transfer) | **Positive hCG** | **Clinical outcome** |
| --- | --- | --- | --- | --- | --- | --- | --- | --- |
| **1** | **P1** | 26 | 5 | 1 E + 4 I | 30.00% (9/30) | 2 (D2) + 1 (D3) | 1 | LB |
|  | **P2** | 36 | 5 | 4 E + 1 I | 7.69% (5/65) | 1 (D2) + 2 (D3) | 1 | LB |
|  | **P3** | 29 | 1 | I | 0.00% (0/10) | - | - | - |
|  | **P4** | 32 | 2 | 1 E + 1 I | 10.00% (1/10) | 0 | - | - |
|  | **P5** | 33 | 4 | E | 25.00% (13/52) | 2 (D3) + 4 (D4) | 1 | LB |
|  | **P6** | 36 | 3 | E | 26.67% (4/15) | 1 (D2) + 2 (D3) | 0 | - |
|  | **P7** | 39 | 4 | E | 2.47% (2/81) | 1 (D2) | 0 | - |
|  | **P8** | 30 | 1 | E | 0.00% (0/13) | - | - | - |
|  | **P9** | 41 | 2 | E | 0.00% (0/13) | - | - | - |
|  |  |  | 1 | E - Oocyte donor | 0.00% (0/5) | - | - | - |
|  | **P10** | 40 | 1 | E | 0.00% (0/4) | - | - | - |
|  | **P11** | 36 | 2 | 1 E + 1 I | 20.00% (3/15) | 1 (D2) | 0 | - |
|  | **P12** | 31 | 2 | E | 13.33% (2/15) | 2 (D2) | 0 | - |
|  | **P13** | 33 | 3 | E | 15.38% (4/26) | 1 (D3) + 2 (D5) | 0 | - |
|  | **P14** | 29 | 2 | E | 0.00% (0/7) | - | - | - |
|  | **P15** | 40 | 3 | E | 31.25% (5/16) | 5 (D3) | 0 | - |
|  | **P16** | 35 | 2 | I | 5.26% (1/19) | 1 (D5) | 0 | - |
|  | **P17** | 38 | 3 | E | 32.14% (9/28) | 2 (D2) + 3 (D3) | 0 | - |
|  | **P18** | 36 | 5 | E | 25.81% (16/62) | 3 (D3) | 1 | M |
|  | **P19** | 31 | 1 | E | 12.50% (1/8) | 1 (D3) | 0 | - |
|  | **P20** | 30 | 2 | E | 18.75% (3/16) | 2 (D3) | 0 |  |
|  | **P21** | 33 | 1 | E | 0.00% (0/8) | - | - | - |
|  | **P22** | 29 | 4 | I | 25.00% (7/28) | 3 (D3) | 2 | M, LB |
|  | **P23** | 32 | 4 | E | 11.54% (3/26) | 2 (D3) | 1 | LB |
|  | **P24** | 41 | 1 | E | 0.00% (0/9) | - | - | - |
|  | **P25** | 27 | 3 | 1 E + 2 I | 19.23% (5/26) | 1 (D5) | 0 | - |
|  | **P26** | 35 | 1 | E | 0.00% (0/11) | - | - | - |
|  | **P27** | 25 | 2 | 1 E + 1 I | 11.11% (2/18) | 0 | - | - |
|  | **P28** | 36 | 3 | E | 25.00% (8/32) | 3 (D3) + 2 (D5) | 0 | - |
| **2** | **P29** | 26 | 1 | E | 13.33% (2/15) | 1 (D3) | 0 | - |
|  | **P30** | 33 | 3 | E | 5.26% (2/38) | 1 (D3) | 1 | LB |
|  | **P31** | 34 | 1 | E | 18.18% (2/11) | 2 (D3) | 1 | BP |
|  | **P32** | 35 | 1 | E | 25.00% (1/4) | 0 | - | - |
|  | **P33** | 34 | 1 | E | 0.00% (0/5) | 0 | - | - |
|  | **P34** | 33 | 2 | E | 0.00% (0/25) | 0 | - | - |
|  | **P35** | 26 | 2 | E | 6.67% (1/15) | 1 (NA) | - | - |
|  | **P36** | 38 | 2 | E | 19.05% (4/21) | 4 (D3) | 1 | M |
|  | **P37** | 31 | 2 | E | 25.00% (2/8) | 1 (D5) | 0 | - |
|  | **P38** | 34 | 1 | E | 21.43% (3/14) | 1 (D3) | 0 | - |
|  | **P39** | 38 | 3 | E | 25.00% (3/12) | 2 (D3) | 0 | - |
|  | **P40** | 35 | 2 | E | 33.33% (2/6) | 2 (D3) | 0 | - |
|  | **P41** | 24 | 2 | E | 0.00% (0/14) | - | - | - |
|  | **P42** | 35 | 1 | E | 28.57% (2/7) | 0 | - | - |
|  | **P43** | 41 | 3 | E | 0.00% (0/10) | - | - | - |
|  | **P44** | 28 | 3 | E | 20.00% (7/35) | 1 (D3) + 2 (D5) | 1 | LB |
|  | **P45** | 31 | 1 | E | 12.50% (1/8) | 1 (D5) | 0 | - |
|  | **P46** | 33 | 3 | E | 8.70% (4/46) | 1 (D3) | 0 | - |
|  |  |  | 1 | E - Sperm donor^b^ | 70.00% (7/10) | Not transferred | - | - |
|  | **P47** | 31 | 1 | E | 0.00% (0/10) | - | - | - |
|  | **P48** | 33 | 3 | E | 0.00% (0/5) | - | - | - |
|  | **P49** | 34 | 2 | E | 31.25% (5/16) | 1 (D3)+ 1 (D5) | 0 | - |
|  | **P50** | 32 | 1 | E | 9.09% (1/11) | 1 (D3) | 0 | - |
|  | **P51** | 28 | 1 | E | 0.00% (0/4) | - | - | - |
|  | **P52** | 36 | 4 | E | 27.27% (3/11) | 1 (D5) | 0 | - |
|  | **P53** | 34 | 1 | E | 0.00% (0/5) | - | - | - |
|  | **P55** | 29 | 3 | E | 21.74% (5/23) | 3 (D3) | 0 | - |
|  | **TOTAL ICSI cycles**^c^ | 33.06 ± 4.26 | 123 | 110 E + 13 I | 14.61% (153/1047) | 77 | +hCG=8.94% (11/123) | LBR=5.69% (7/123) |

^a^ICSI cycles were performed in external IVF centers (external, E) or at Ghent University Hospital (internal, I) ^b^Sperm donor was used as a diagnostic test and blastocysts derived from this cycle were not transferred. ^c^Only ICSI cycles with patient sperm are included in the total. BP: biochemical pregnancy; D: day; LB: live birth; LBR: live birth rate; M: miscarriage; MII: metaphase II oocyte; 2PN: two pronuclei; +hCG: positive hCG rate.

**Supplementary Table S2 Clinical outcomes after ICSI-AOA cycles using a different protocol than CaCl_2_ injection and double ionomycin exposure in patients with previous total fertilization failure or low fertilization (**≤**33.33%) after ICSI (P1-P55).**

| **Cohort** | **Patient** | **ICSI-AOA cycles, n** | **External** (E)/ **Internal** (I) **cycles^a^** | **Fertilization rate** (2PN/MII) | **N embryos transferred**  (Day of transfer) | **Positive hCG** | **Clinical outcome** |
| --- | --- | --- | --- | --- | --- | --- | --- |
| **1** | **P1** | 2 | I - mechanical | 33.33% (5/15) | 2 (D2) | 0 | - |
|  | **P2** | 2 | I - electrical | 26.92% (7/26) | 5 (D2) | 0 | - |
|  | **P8** | 1 | E - Ca^2+^ ionophore, NA^b^ | 0.00% (0/5) | - | - | - |
|  | **P12** | 1 | E - Ca^2+^ ionophore, NA^b^ | 33.33% (1/3) | 1 (D2) | 0 | - |
|  | **P26** | 1 | E - Ca^2+^ ionophore, Calcimycin^c^ | 0.00% (0/11) | - | - | - |
|  | **P42** | 1 | E -Ca^2+^ ionophore, NA^b^ | 0.00% (0/7) | - | - | - |
|  | **P45** | 1 | E - Ca^2+^ ionophore, NA^b^ | 11.11% (1/9) | 1 (D5) | 0 | - |
|  | **P46** | 1 | E - Ca^2+^ ionophore, Calcimycin^c^ | 0.00% (0/11) | - | - | - |
|  |  | 1 | E - Ca^2+^ ionophore, Ionomycin | 10.00% (1/10) | 0 | - | - |
|  | **P47** | 1 | E - Ca^2+^ ionophore, Calcimycin^c^ | 0.00% (0/6) | - | - | - |
|  | **P54** | 2 | E - Ca^2+^ ionophore, NA^b^ | 14.29% (2/14) | 1 (D5) | 0 | - |

^a^ICSI-AOA cycles were performed in external IVF centers or at Ghent University Hospital ^b^AOA is performed with calcium ionophore, but data on the specific ionophore is not available (NA). ^c^AOA protocol according to manufacturer (CultActive, Sierksdorf,  Gynemed). D: day; MII: metaphase II oocyte; 2PN: two pronuclei.

**Supplementary Table S3 Sperm sample characteristics and MOAT results of patients with total fertilization failure or low fertilization (**≤**33.33%) after ICSI (P1-P55).**

| **Cohort** | **Patient** | **Sperm volume**  (ml) | **Sperm concentration** (M/ml) | **Sperm motility**  (a+b) % | **Morphology**  (Normal shape, %) | **MOAT group** | **MOAT Activation rate** (2cell/MII) |
| --- | --- | --- | --- | --- | --- | --- | --- |
| **1** | **P1** | 3.00 | 10.00 | 1 | 0 | 2 | 33.33% (7/21) |
|  | **P2** | 5.50 | 72.00 | 48 | 8 | 1 | 19.23% (5/26) |
|  | **P3** | 2.70 | 0.10 | 0 | 0 | 2 | 40.00% (8/20) |
|  | **P4** | 4.00 | 0.50 | 0 | 0 | 2 | 77.78% (21/27) |
|  | **P5** | 3.20 | 11.00 | 40 | 0 | 2 | 65.63% (21/32) |
|  | **P6** | 5.00 | 10.20 | 10 | 2 | 2 | 51.35% (19/37) |
|  | **P7** | 4.30 | 3.00 | 16 | 0 | 2 | 83.87% (26/31) |
|  | **P8** | 3.50 | 17.00 | 50 | 6 | 2 | 48.48% (16/33) |
|  | **P9** | 2.50 | 91.00 | 48 | 8 | 3 | 83.33% (20/24) |
|  | **P10** | 2.50 | 105.27 | 43.8 | 7 | 3 | 86.67% (26/30) |
|  | **P11** | 3.70 | 30.21 | 43.3 | 4 | 3 | 86.96% (20/23) |
|  | **P12** | 2.80 | 3.00 | 24 | 2 | 2 | 65.63% (21/32) |
|  | **P13** | 3.20 | 3.00 | 28 | 2 | 2 | 76.00% (19/25) |
|  | **P14** | 5.20 | 15.00 | 2 | 0 | 1-2 | 22.22% (6/27) |
|  | **P15** | 5.10 | 5.00 | 11 | 0 | 2 | 50.00% (7/14) |
|  | **P16** | 3.50 | 24.26 | 19.9 | 4 | 2 | 83.87% (26/31) |
|  | **P17** | 2.90 | 31.21 | 28.6 | 6 | 2 | 72.41% (21/29) |
|  | **P18** | 1.00 | 110.00 | 15 | 2 | 2 | 54.17% (13/24) |
|  | **P19** | 2.50 | 12.95 | 4 | 2 | 2 | 74.07% (20/27) |
|  | **P20** | 3.50 | 2.00 | 23 | 0 | 2 | 63.64% (14/22) |
|  | **P21** | 4.50 | 140.00 | 57 | 1 | 3 | 89.47% (17/19) |
|  | **P22** | 4.00 | 0.10 | 28 | 0 | 2 | 66.67% (18/27) |
|  | **P23** | 5.50 | 11.09 | 18.2 | 1 | 2 | 78.57% (22/28) |
|  | **P24** | 3.46 | 48.56 | 40.9 | 3 | NA^a^ | NA^a^ |
|  | **P25** | 3.20 | 10.88 | 15.6 | NA | 2 | 73.08% (19/26) |
|  | **P26** | 3.00 | 57.33 | 37.5 | NA | 3 | 89.66% (26/29) |
|  | **P27** | 4.58 | 8.59 | 19.3 | 2 | 2 | 52.00% (13/25) |
|  | **P28** | 3.26 | 10.01 | 9.3 | 0 | 2 | 44.00% (11/25) |
| **2** | **P29** | 4.85 | 34.03 | 15.20 | 0 | 1-2 | 25.81% (8/31) |
|  | **P30** | 2.43 | 13.56 | 14.50 | 1 | 2 | 46.67% (14/30) |
|  | **P31** | 3.18 | 46.11 | 54.80 | 4 | 3 | 87.88% (29/33) |
|  | **P32** | 2.56 | 46.26 | 15.30 | 2 | 3 | 92.86% (26/28) |
|  | **P33** | 3.57 | 3.10 | 28.00 | 2 | 2 | 65.52% (19/29) |
|  | **P34** | 2.68 | 64.88 | 22.40 | 4 | 3 | 87.50% (28/32) |
|  | **P35** | 6.00 | 15.64 | 6.40 | 2 | 2 | 79.17% (19/24) |
|  | **P36** | 1.00 | 1.70 | 47.00 | NA | 3 | 100.00% (30/30) |
|  | **P37** | 2.72 | 3.30 | 22.00 | 1 | 3 | 87.50% (21/24) |
|  | **P38** | 4.47 | 18.14 | 10.00 | 2 | 3 | 88.89% (24/27) |
|  | **P39** | 2.45 | 9.82 | 42.60 | 3 | 3 | 96.30% (26/27) |
|  | **P40** | 2.80 | 1.90 | 40.00 | 0 | 3 | 89.66% (26/29) |
|  | **P41** | 4.93 | 4.86 | 11.10 | 1 | 2 | 53.85% (14/26) |
|  | **P42** | 2.61 | 5.40 | 22.00 | 0 | 3 | 87.10% (27/31) |
|  | **P43** | 2.50 | 40.78 | 22.70 | 3 | 3 | 92.31% (36/39) |
|  | **P44** | 3.49 | 7.16 | 57.00 | 2 | 2 | 80.00% (20/25) |
|  | **P45** | 2.77 | 4.14 | 11.10 | 0 | 3 | 96.43% (27/28) |
|  | **P46** | 2.71 | 52.76 | 25.40 | 0 | 1-2 | 25.81% (8/31) |
|  | **P47** | 5.13 | 1.40 | 11.00 | 0 | 2 | 81.82% (18/22) |
|  | **P48** | 3.4 | 32.20 | 7.90 | 3 | 3 | 100.00% (36/36) |
|  | **P49** | 5.5 | 32.35 | 45.70 | 3 | 3 | 97.14% (34/35) |
|  | **P50** | 2.02 | 24.25 | 11.30 | 0 | 2 | 60.61% (20/33) |
|  | **P51** | 3.93 | 10.30 | 1.20 | 0 | 3 | 90.70% (39/43) |
|  | **P52** | 5.5 | 1.35 | 23.10 | 1 | 3 | 93.48% (43/46) |
|  | **P53** | 6.5 | 0.80 | 35.00 | NA | 2 | 83.02% (44/53) |
|  | **P54** | 3.4 | 32.8 | 58.00 | 7 | 1 | 7.14% (3/42) |
|  | **P55** | 3.9 | 0.40 | 20.00 | 0 | 2 | 83.33% (35/42) |
|  | **Low reference limits*** | 1.40 | 16.00 | 30.00 | 4 |  |  |

^a^MOAT not done due to ethical objection *Based on WHO laboratory manual for the examination and processing of human semen. Sixth Edition. a+b: rapid and slow progressive sperm motility; MOAT: mouse oocyte activation test; MII: metaphase II oocyte; NA: not available.

**Supplementary Table S4 Clinical outcomes after ICSI-AOA cycles, using CaCl_2_ injection and double ionomycin exposure, in patients with previous total fertilization failure or low fertilization (**≤**33.33%) after ICSI (P1-P55).**

| **Cohort** | **Patient** | **Age** (in the last ICSI-AOA cycle) | **ICSI-AOA cycles^a^, n** | **Fertilization rate** (2PN/MII) | **Blastocyst rate** (blastocyst/2PN) | **Good quality blastocyst rate** (good quality blastocyst/2PN) | **N cryopreserved embryos on D5** (otherwise indicated) | **N fresh or thawed embryos transferred on D5** (otherwise indicated) | **Positive hCG** | **Clinical outcome** |
| --- | --- | --- | --- | --- | --- | --- | --- | --- | --- | --- |
| **1** | **P1** | 38 | 1 | 100.00% (8/8) | NA | NA | 4 (D2) | 1 (D2) | 1 | LB |
|  | **P2** | 38 | 1 | 40.00% (8/20) | NA | NA | 3 (D3) | 5 (D3) | 1 | LB |
|  | **P3** | 35 | 8 | 68.25% (43/63) | NA | NA | 12 (D2) + 7 (D3) | 7 (D3) + 8 (D2) | 1 | ND |
|  | **P4** | 32 | 1 | 83.33% (5/6) | NA | NA | 1 (D3) | 1 (D3) | 0 | - |
|  | **P5** | 34 | 1 | 93.75% (15/16) | 13.33% (2) | 0.00% (0) | 0 | 2 | 0 | - |
|  | **P6** | 38 | 1 | 16.67% (1/6) | 0.00% (0) | 0.00% (0) | - | - | - | - |
|  | **P7** | 39 | 1 | 80.00% (12/15) | 50.00% (6) | 16.67% (2) | 3 | 2 | 1 | LB |
|  | **P8** | 31 | 1 | 100.00% (13/13) | 15.38% (2) | 0.00% (0) | 0 | 2 | 0 | - |
|  | **P9** | 42 | 2 | 30.77% (4/13) | 0.00% (0) | 0.00% (0) | - | - | - | - |
|  | **P10** | 41 | 1 | 75.00% (3/4) | 33.33% (1) | 0.00% (0) | 0 | 1 | 0 | - |
|  | **P11** | 36 | 1 | 80.00% (4/5) | 100.00% (4) | 25.00% (1) | 2 | 1 | 1 | LB |
|  | **P12** | 33 | 2 | 63.16% (12/19) | 50.00% (6) | 16.67% (2) | 4 ^c^ | 1 | NA | NA |
|  | **P13** | 35 | 1 | 25.00% (1/4) | 100.00% (1) | 100.00% (1) | 0 | 1 | 0 | - |
|  | **P14** | 29 | 1 | 60.00% (3/5) | 0.00% (0) | 0.00% (0) | - | - | - | - |
|  | **P15** | 41 | 3 | 28.57% (2/7) | 100.00% (2) | 50.00% (1) | 1 | 1 | 1 | BP |
|  | **P16** | 35 | 1 | 15.38% (2/13) | 100.00% (2) | 0.00% (0) | 2 | 0 | - | - |
|  | **P17** | 40 | 1 | 14.29% (1/7) | 0.00% (0) | 0.00% (0) | - | - | - | - |
|  | **P18** | 36 | 1 | 61.54% (8/13) | 62.50% (5) | 12.50% (1) | 3 | 2 | 2 | BP, LB |
|  | **P19** | 34 | 1 | 71.43% (5/7) | 20.00% (1) | 0.00% (0) | 0 | 0 | - | - |
|  |  |  | 1^b^ | 83.33% (5/6) | 0.00% (0) | 0.00% (0) | - | - | - | - |
|  | **P20** | 31 | 1 | 61.54% (8/13) | 87.50% (7) | 25.00% (2) | 6 | 5 | 2 | 2xLB |
|  | **P21** | 33 | 1 | 76.47% (13/17) | 38.46% (5) | 15.38% (2) | 2 | 1 | 1 | LB |
|  | **P22** | 30 | 1 | 40.00% (2/5) | 0.00% (0) | 0.00% (0) | - | - | - | - |
|  | **P23** | 34 | 2 | 62.50% (10/16) | 80.00% (8) | 10.00% (1) | 1 | 3 | 1 | M |
|  | **P24** | 42 | 1 | 25.00% (1/4) | 100.00% (1) | 0.00% (0) | 0 | 1 | 0 | - |
|  | **P25** | 27 | 1 | 26.67% (4/15) | 100.00% (4) | 100.00% (4) | 3 | 3 | 2 | 2xLB |
|  | **P26** | 37 | 1 | 77.78% (14/18) | 71.43% (10) | 21.43% (3) | 6 | 5 | 1 | BP |
|  | **P27** | 35 | 2 | 50.00% (5/10) | 40.00% (2) | 20.00% (1) | 0 | 2 | 0 | - |
|  | **P28** | 38 | 2 | 57.89% (11/19) | 36.36% (4) | 27.27% (3) | 1 | 3 | 2 | LB, BP |
| **2** | **P29** | 27 | 1 | 81.82% (9/11) | 66.67% (6) | 55.56% (5) | 6 | 3 | 2 | 2xLB |
|  | **P30** | 35 | 1 | 92.31% (12/13) | 91.67% (11) | 41.67% (5) | 7 | 6 | 1 | LB |
|  | **P31** | 36 | 3 | 72.22% (13/18) | 92.31% (12) | 23.08% (3) | 9 | 9 | 0 | - |
|  | **P32** | 39 | 4 | 45.00% (9/20) | 88.89% (8) | 66.67% (6) | 2 | 5 | 1 | LB |
|  |  |  | 1^b^ | 80.00% (4/5) | 75.00% (3) | 25.00% (1) | 0 | 1 | 1 | LB |
|  | **P33** | 35 | 1 | 75.00% (6/8) | 66.67% (4) | 66.67% (4) | 3 | 2 | 2 | LB, BP |
|  | **P34** | 34 | 1 | 100.00% (11/11) | 72.73% (8) | 27.27% (3) | 5 | 2 | 2 | LB, BP |
|  | **P35** | 27 | 2 | 45.16% (14/31) | 50.00% (7) | 0.00% (0) | 1 | 2 | 1 | LB |
|  | **P36** | 40 | 1 | 100.00% (7/7) | 71.43% (5) | 42.86% (3) | 4^c^ | 1 | 0 | - |
|  | **P37** | 33 | 2 | 85.71% (18/21) | 72.22% (13) | 44.44% (8) | 10^c^ | 3 | 1 | BP |
|  | **P38** | 35 | 2 | 73.33% (22/30) | 45.45% (10) | 4.55% (1) | 1 | 1 | 0 | - |
|  | **P39** | 38 | 1 | 50.00% (6/12) | 100.00% (6) | 33.33% (2) | 5 | 1 | 1 | LB |
|  | **P40** | 36 | 1 | 77.78% (7/9) | 28.57% (2) | 0.00% (0) | 0 | 1 | 1 | LB |
|  | **P41** | 25 | 1 | 100.00% (8/8) | 50.00% (4) | 37.50% (3) | 3 | 1 | 1 | LB |
|  | **P42** | 35 | 1 | 30.00% (3/10) | 66.67% (2) | 33.33% (1) | 1 | 1 | 0 | - |
|  | **P43** | 42 | 2 | 80.00% (4/5) | 50.00% (2) | 0.00% (0) | 0 | 1 | 0 | - |
|  | **P44** | 29 | 1 | 66.67% (6/9) | 83.33% (5) | 33.33% (2) | 2 | 1 | 1 | LB |
|  | **P45** | 31 | 1 | 61.54% (8/13) | 100.00% (8) | 25.00% (2) | 5^d^ | 0 | NA | NA |
|  | **P46** | 34 | 1 | 26.32% (5/19) | 60.00% (3) | 60.00% (3) | 3 | 2 | 1 | Ong |
|  | **P47** | 33 | 1 | 28.57% (2/7) | 100.00% (2) | 50.00% (1) | 0 | 1 | 0 | - |
|  | **P48** | 34 | 2 | 84.62% (11/13) | 18.18% (2) | 0.00% (0) | 0 | 1 | 0 | - |
|  | **P49** | 35 | 1 | 37.50% (3/8) | 100.00% (3) | 100.00% (3) | 2 | 1 | 1 | LB |
|  | **P50** | 33 | 1 | 71.43% (5/7) | 60.00% (3) | 20.00% (1) | 0 | 1 | 0 | - |
|  | **P51** | 28 | 1 | 90.00% (9/10) | 88.89% (8) | 66.67% (6) | 8 | 3 | 2 | BP, Ong |
|  | **P52** | 35 | 1 | 20.00% (2/10) | 100.00% (2) | 0.00% (0) | 1 | 1 | 0 | - |
|  | **P53** | 35 | 1 | 90.00% (9/10) | 77.78% (7) | 33.33% (3) | 6 | 1 | 1 | BP |
|  | **P54** | 36 | 1 | 87.50% (7/8) | 85.71% (6) | 42.86% (3) | 4 | 1 | 1 | Ong |
|  | **P55** | 30 | 1 | 68.75% (11/16) | 54.55% (6) | 27.27% (3) | 3 | 1 | 1 | Ong |
|  | **TOTAL** | 34.62 ± 4.07 | 81 | 63.41% (454/716) | 59.23% (231/390)^e^ | 24.62% (96/390)^e^ | 155 | 112 | +hCG =48.15% (39/81) | LBR =29.63% (24/81) |

^a^All ICSI-AOA cycles were performed in Ghent University Hospital with CaCl_2_ injection and double ionomycin exposure. ^b^Oocyte donation was used in these ICSI-AOA cycles. ^c^PGT was performed in these cycles, thus blastocysts that carried the corresponding mutation were not used. ^d^Cryopreserved embryos were transferred to an external clinic. Information regarding embryo transfers is missing. ^e^Blastocyst rate and good quality blastocyst rate is calculated over the fertilized oocytes in cycles with embryo culture to blastocyst state. BP: biochemical pregnancy; D: day; LB: live birth; LBR: live birth rate; M: miscarriage; MII: metaphase II oocyte; ND: neonatal death; Ong: ongoing pregnancy; 2PN: two pronuclei; +hCG: positive hCG rate.

**Supplementary Table S5 Benign and pathogenic criteria used for variant classification.**

| **Patients** | **Gene** | **c.DNA change** | **aa change** | **RefSNP** | **Bening classification criteria^a^** | **Pathogenic classification criteria^a^** | **ClinGen Bayesian**  **Classification**  (after functional testing)^b^ |
| --- | --- | --- | --- | --- | --- | --- | --- |
| P32 | PLCZ1 | c.221T>C | p.Ile74Thr | rs145549980 | BP4, **BS3_BP** | PM2, PP4_PM | VUS (tepid; p = 0.5) |
| P26 | PLCZ1 | c.280C>T | p.Gln94* | [rs138801851](https://varsome.com/variant/hg38/rs138801851?&annotation-mode=germline) | - | PM2, PP4_PM, PVS1_PS2_PM, **PS3_PP** | LP (p = 0.949) |
| P9 | PLCZ1 | c.422G>A | p.Arg141His | rs202034240 | BP4 | PM2, PP4_PM, **PS3_PP** | VUS (warm; p = 0.675) |
| P2, P21, P34, P35, P44 | PLCZ1 | c.698A>T | p.His233Leu | [rs200061726](https://www.ncbi.nlm.nih.gov/snp/rs200061726) | BP4 | PM2, PP4_PM, PP5, PM5_PM, PM1_PM, **PS3_PP,** PP1 | P (p = 0.994) |
| P2 | PLCZ1 | c.964A>T | p.Lys322* | NA | - | PM2, PP4_PM, PVS1_PS1_PM, **PS3_PP** | LP (p = 0.949) |
| P36 | PLCZ1 | c.1136T>C | p.Ile379Thr | rs201548309 | BP4 | PM2, PP4_PM, PM1_PM, **PS3_PP** | LP (p = 0.9) |
| P3, P4, P7, P15, P18, P20, P23, P26 | PLCZ1 | c.1499C>T | p.Ser500Leu | rs10505830 | BP4, BS3_BP | PS4, PP4_PM, **PS3_PP** | LP (p = 0.9) |
| P32 | ACTL7A | c.547T>C | p.Tyr183His | rs41278345 | **BS3_BP** | PM2, PP4_PM, PP3 | VUS (warm, p=0.675) |
| P46 | ACTL7A | c.640G>A | p.Gly214Ser | rs41278347 | - | PP4_PM, PP3, **PS3_PP** | VUS (tepid; p = 0.5) |
| P54 | ACTL7A | c.657G>A | p.Val219= | rs3739693 | BP4, BP7 | PP4_PM, **PS3_PP** | VUS (cold; p = 0.188) |
| P54 | ACTL7A | c.1018G>A | p.Val340Met | rs7872077 | **BS3_BP** | PP4_PM, **PS3_PP** | VUS (cool; p = 0.325) |
| P29 | ACTL7A | c.1088dup | p.Ser364Glnfs*9 | rs752334307 | - | PM2, PP4_PM, PSV1_PS2_PM, PS1, **PS3_PP** | P (p=0.997) |
| P46 | ACTL7A | c.1117C>T | p.Arg373Cys | rs775405375 | - | PM2, PP4_PM, PP5, PP3, PM5_PM, PM1_PM, **PS3_PP** | P (p = 0.994) |
| P34 | ACTL9 | c.812G>C | p.Arg271Pro | rs73507819 | BP4, **BS3_BP** | PP4_PM | VUS (cool; p = 0.188) |

^a^The definition for each criteria can be found in Richards *et al.*, 2015 . ^b^Variant classification was performed using the VCT 2020.2 tool from the Center of Medical Genetics Ghent. Final classification is reported according to the ACMG/AMP guidelines with Bayesian framework (Tavtigian *et al.*, 2018) considering functional testing performed in this study. In bold criteria selected after functional testing. LP = likely pathogenic; p indicates posterior probability; P = pathogenic, VUS = variant of uncertain significance.

**Supplementary Table S6 Functional analysis of *ACTL7A* and *ACTL9* variants identified in patients who previously suffered from poor fertilization after ICSI.**

|  |  |  | **ACTL7A immunostaining** | | | |  | **TEM analysis** | |
| --- | --- | --- | --- | --- | --- | --- | --- | --- | --- |
| **Cohort** | **Patient** | **Identified variant**  (gene, aa change, zygosity) | **N sperm cells with intact acrosome** | Invisible ACTL7A expression (%) | Weak ACTL7A expression (%) | Obvious ACTL7A expression (%) | **ACTL7A expression** (weak + obvious, %) | **N sperm cells with intact acrosome** | **Detached acrosome** (%) |
| **2** | **P29** | ACTL7A, p.(Ser364GlnfsTer9), Hom | 57 | 75.44% (43) | 10.53%  (6) | 14.04%  (8) | 24.56%  (14)** | 28 | 89.29%  (25)** |
|  | **P32** | ACTL7A, p.(Tyr183His), Het  PLCZ1, p.(Ile74Thr), Het | 64 | 32.81% (21) | 32.81% (21) | 34.38% (22) | 67.19%  (43) | 26 | 38.46%  (10) |
|  | **P34** | ACTL9, p.(Arg271Pro), Het  PLCZ1, p.(His233Leu), Het | NA | NA | NA | NA | NA | 29 | 44.83%  (13) |
|  | **P46** | ACTL7A, p.(Gly214Ser), Hom  ACTL7A, p.(Arg373Cys), Hom | 51 | 84.31% (43) | 15.69%  (8) | 0.00%  (0) | 15.69%  (8)** | 22 | 63.64%  (14)* |
|  | **P54** | ACTL7A, p.(Val340Met), Het  ACTL7A, p.(Val219=), Het | 68 | 41.18% (28) | 14.71% (10) | 44.12% (30) | 58.82%  (40) | 24 | 62.50%  (15)* |
|  | **C** | - | 75 | 33.33% (25) | 44.00% (33) | 22.67% (17) | 66.67%  (50) | 45 | 33.33% (15) |

Patient results were compared to the control group using Fisher’s exact test. *p<0.05 **p-value<0.0001. C: control; Hom: homozygous; Het: heterozygous; NA: not available; P: patient; TEM: transmission electron microscopy.

**Supplementary Table S7 Obstetrical and neonatal outcomes of babies born after ICSI-AOA cycles in patients with previous total fertilization failure or low fertilization (**≤**33.33%) after ICSI.**

| **Parameter** | **AOA** |
| --- | --- |
| Live births | 25^a^ |
| Obstetrical outcomes |  |
| Preterm delivery (<37 weeks) | 3/24 |
| Vaginal delivery | 16 |
| Cesarean section | 3 |
| Not available | 5 |
| Neonatal outcomes |  |
| Mean birth weight (g) | 3262.9 ± 736.7 |
| Birth weight <2500 g | 1 |
| Mean Apgar score at 3min | 9.06 ± 2.02 |
| Apgar score <7 at 3min | 1 |
| Perinatal mortality | 1^a^ |
| Sex |  |
| Male | 13 |
| Female | 12 |

All children were born without malformations, except one child that was born with only one normal functioning kidney. The birth weight of 4 children and Apgar score of 9 children are not available. ^a^One baby died after birth.
