# Supplementary material for: High rate of detected variants in male PLCZ1 and ACTL7A genes causing failed fertilization after ICSI
Source: Hum Reprod Open. 2024 Sep 28;2024(4):hoae057. doi: 10.1093/hropen/hoae057 (PMC11479693; doi:10.1093/hropen/hoae057)
Supplement: hoae057_Supplementary_Data [file hoae057_supplementary_data.zip › HRO-24-0042-R2-SuppFigs1-4.docx.docx]

***PLCZ1* missense variants**

**c.221T>C/p.Ile74Thr**


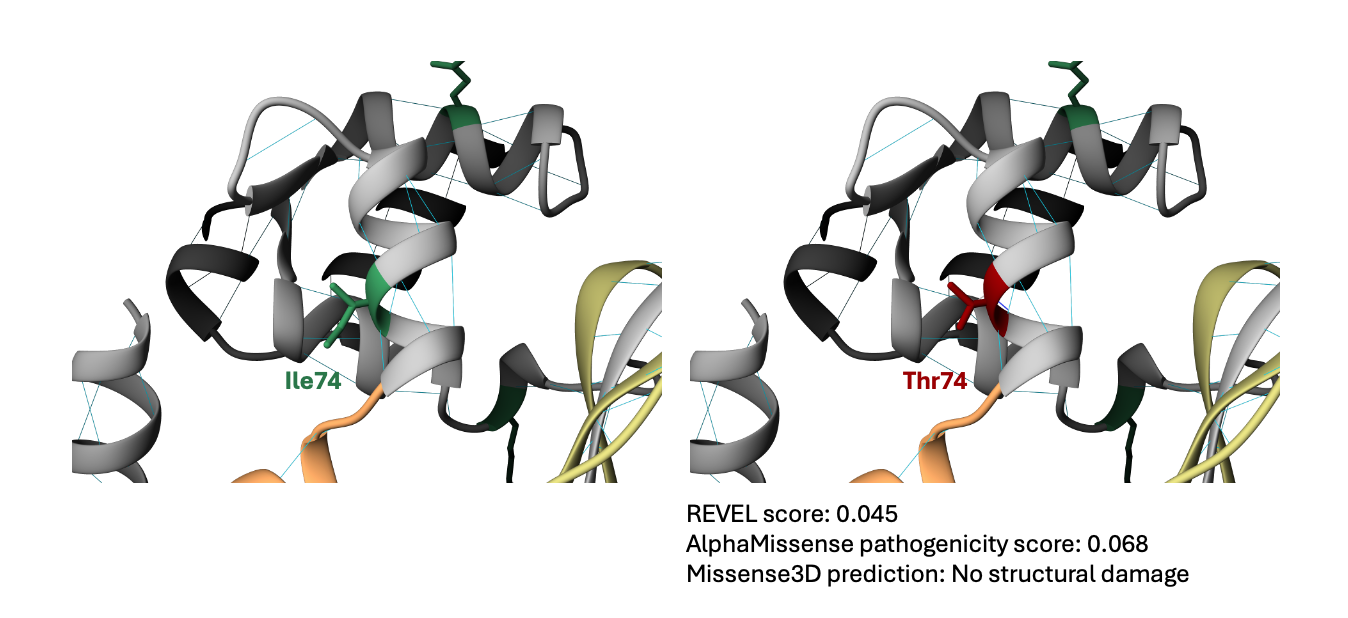


**c.422G>A/p.Arg141His**


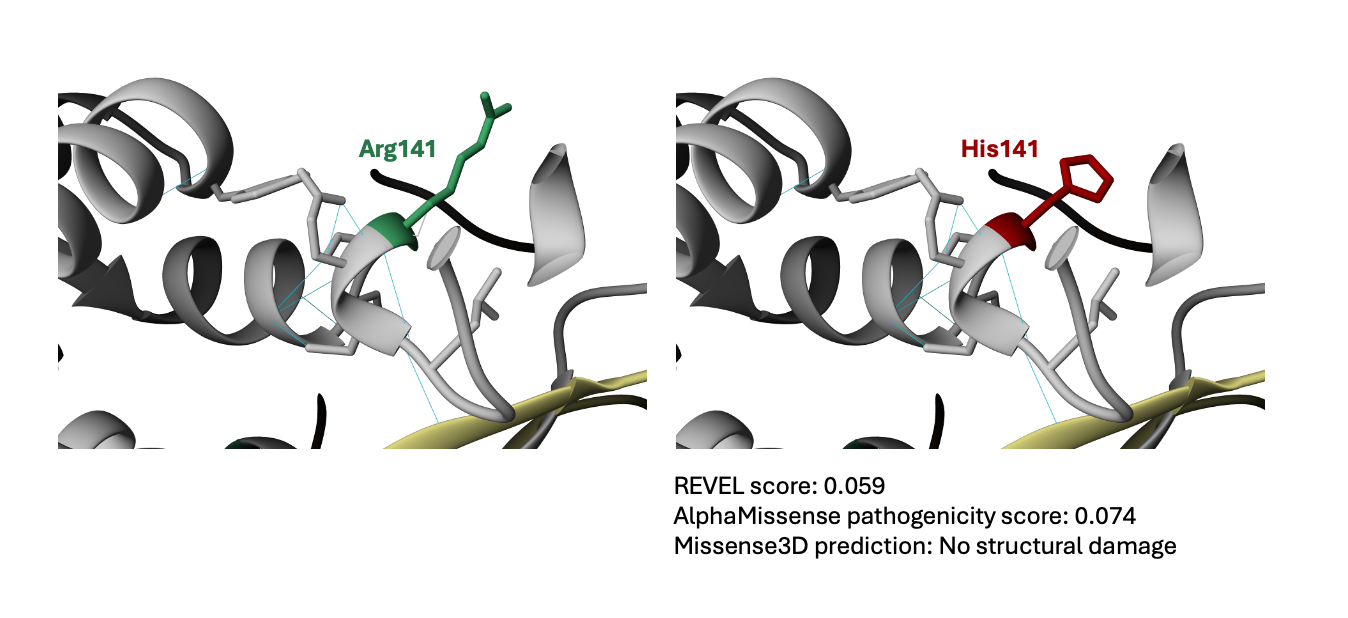


**c.698A>T/p.His233Leu**


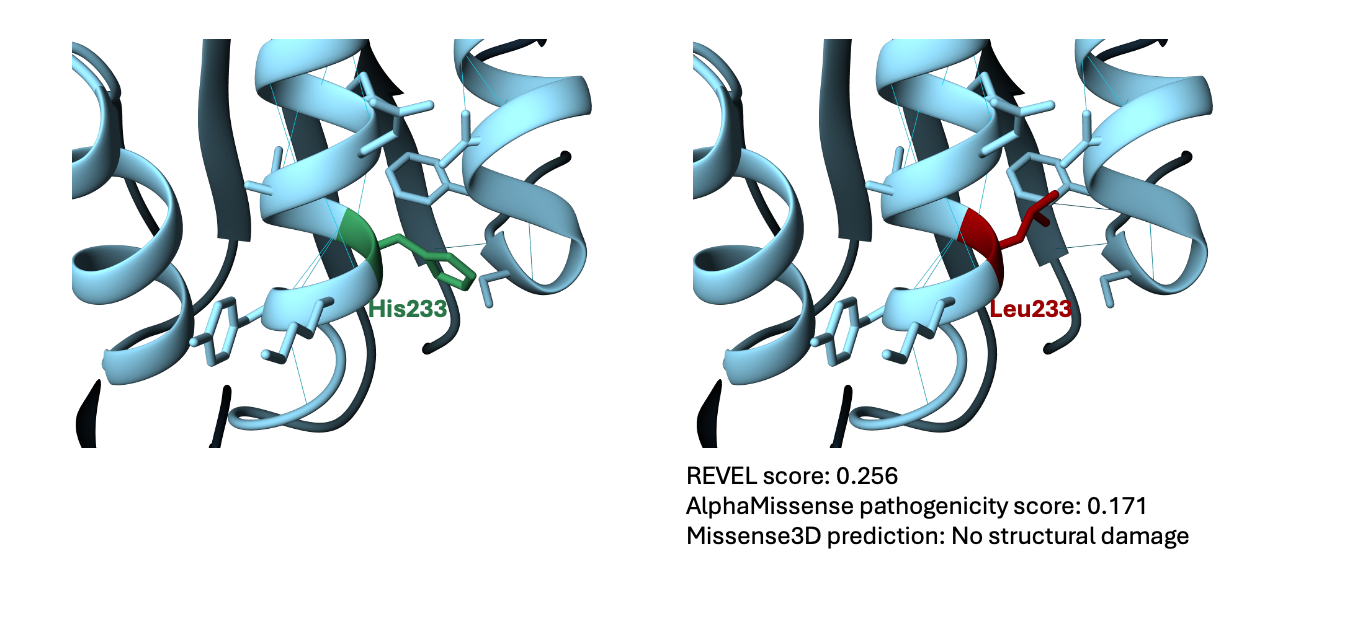


**c.1136T>C/p.Ile379Thr**


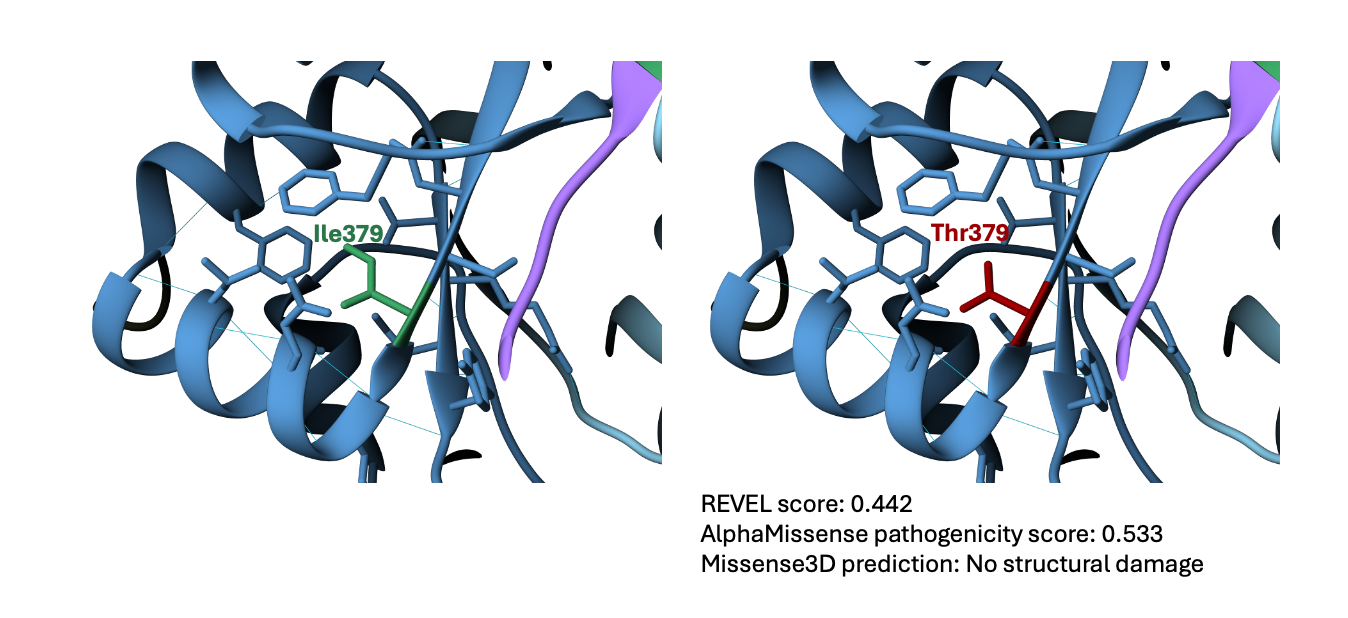


**c.1499C>T/p.Ser500Leu**


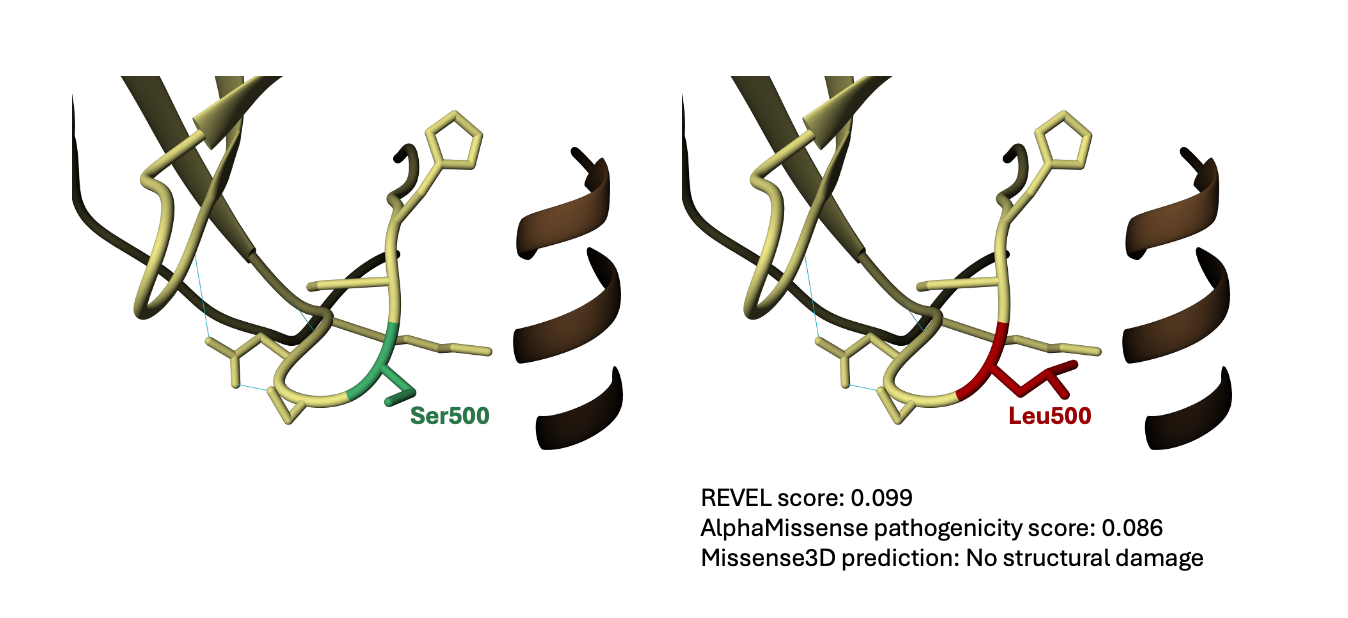


***ACTL7A* missense variants**

**c.547T>C/p.Tyr183His**


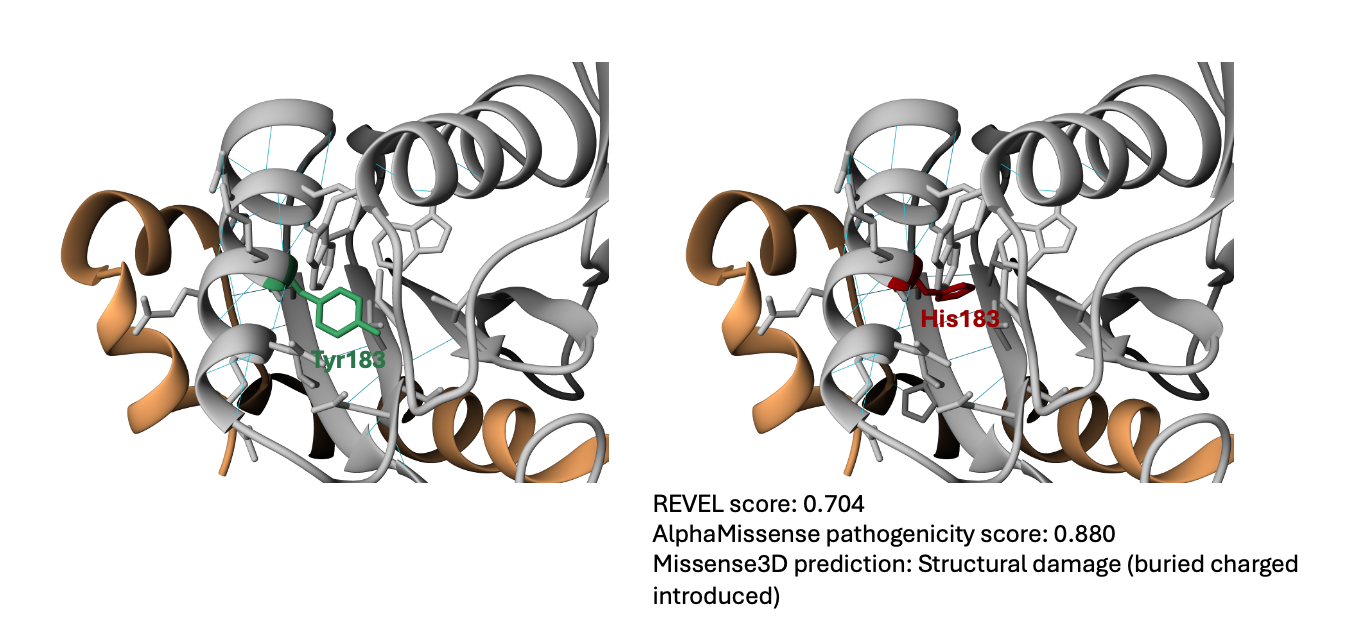


**c.640G>A/p.Gly214Ser**


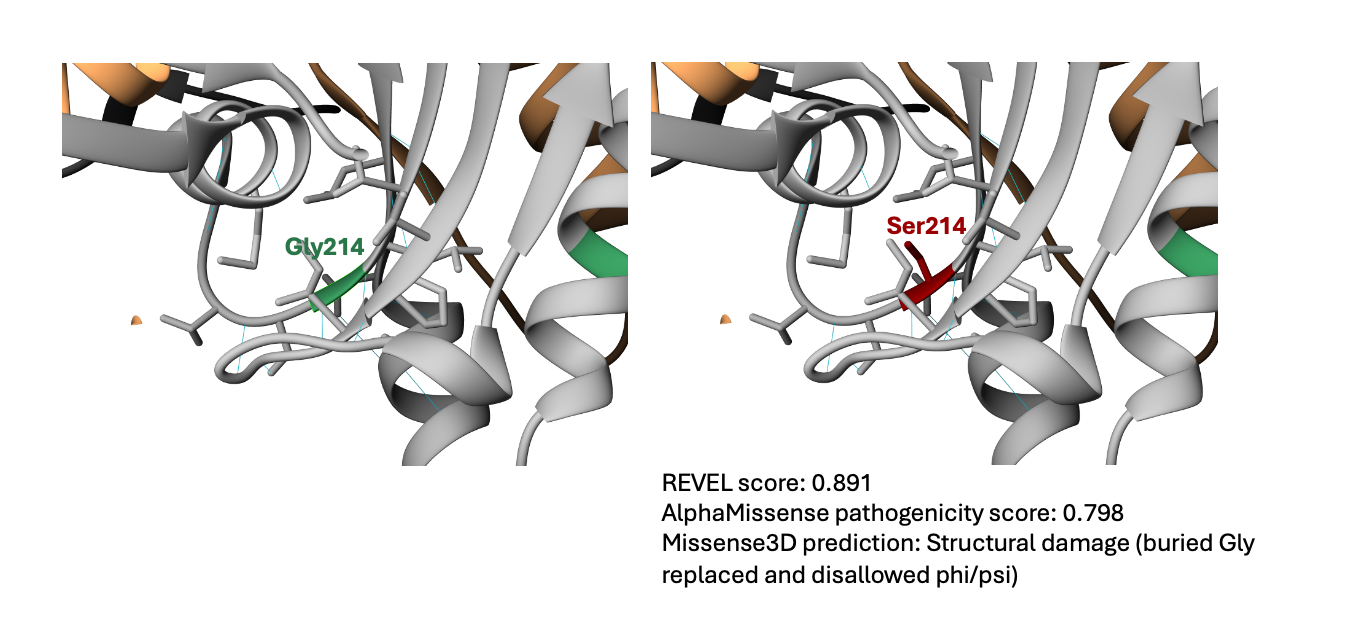


**c.1018G>A/p.Val340Met**


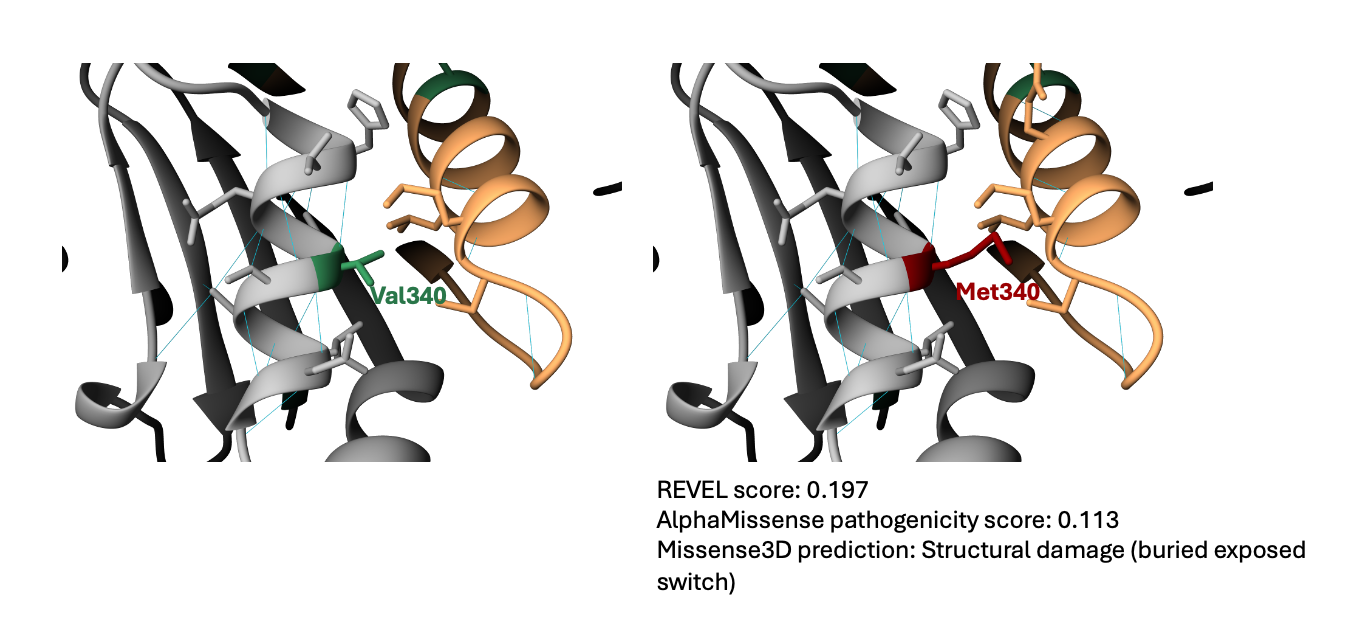


**c.1117C>T/p.Arg373Cys**


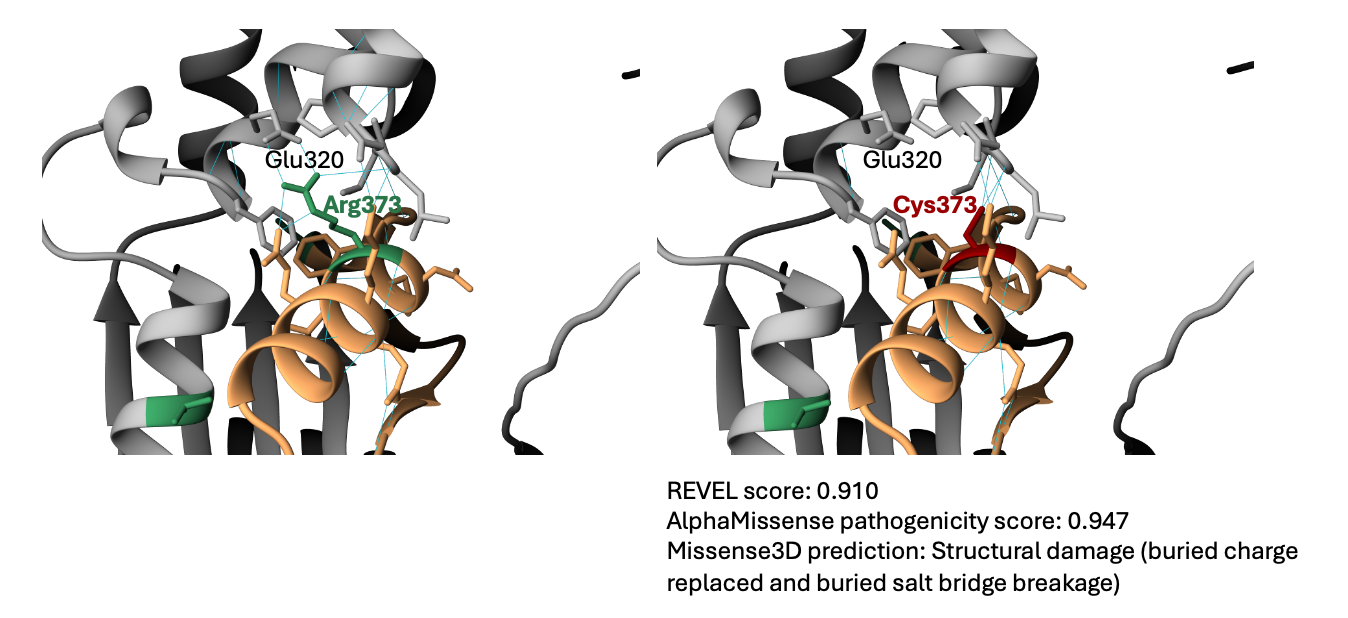


***ACTL9* missense variants**

**c.812G>C/p.Arg271Pro**


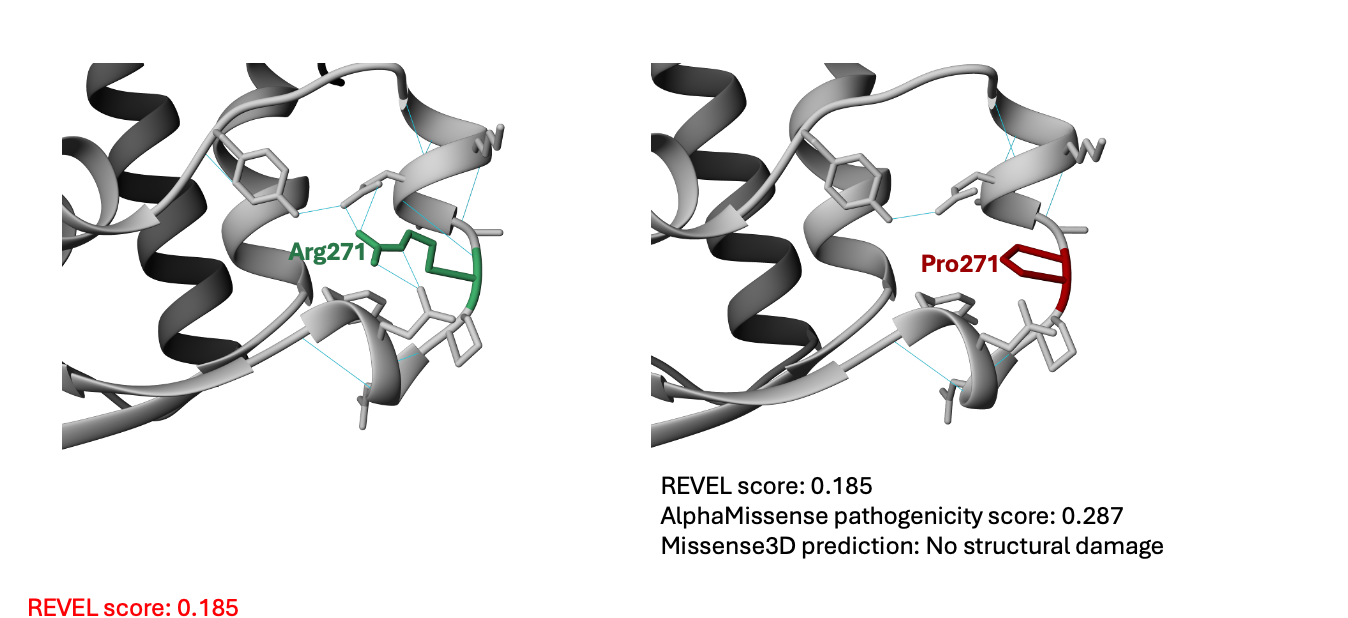


**Supplementary Figure S1 Effect of detected *PLCZ1, ACTL7A,* and *ACTL9* missense variants on protein structure.** Different *in silico* tools (REVEL score, AlphaMissense and Missense3D) were used to assess the impact of missense variants on protein composition. Predicted three-dimensional protein structures were obtained from AlphaFold. Figures were prepared with UCSF Chimera. REVEL (rare exome variant ensemble learner) is an ensemble method that integrates the predictions from several individual tools, including MutPred, FATHMM, VEST, PolyPhen, SIFT, PROVEAN, MutationAssessor, MutationTaster, LRT, GERP, SiPhy, phyloP, and phastCons. A REVEL score higher than 0,5 indicates pathogenicity. AlphaMissense is a machine learning model that predicts the pathogenicity of missense variants based on dual learned protein structure and evolutionary features. AlphaMissense classifies variants with a pathogenicity score as follows: 0-0.34 (likely benign), 0.34-0.564 (uncertain), and 0.564-1 (likely pathogenic). Missense3D is a computational tool that predicts the impact of missense variants on predicted three-dimensional structure of the protein. Missense3D gives a prediction based on 17 different structural features of disease-associated substitutions. Buried charged introduced/replaced: the substitution replaces a buried uncharged/charged residue with a charged/uncharged residue. Buried Gly replaced: the substitution replaces a buried glycine. Disallowed phi/psi: the mutant residue is in an outlier region, while the wild-type residue in the favored or allowed regions. Buried salt bridge breakage: the substitution breaks a salt bridge formed by the wild-type residue which was buried. Buried exposed switch: The substitution results in a change between buried and exposed state of the target residue.

***PLCZ1* variants**

**c.221T>C/p.Ile74Thr** (chr12:g.18723457A>G)

Identified in a heterozygous state in P32


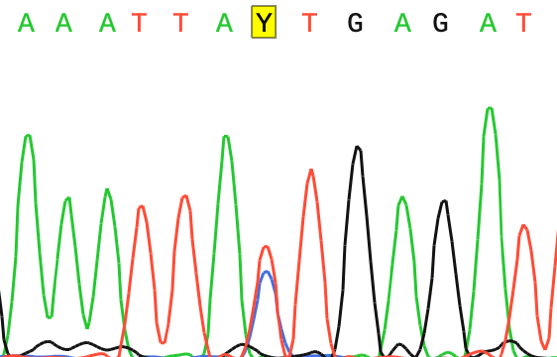


**c.280C>T/p.Gln94*** (chr12:g.18723398G>A)

Identified in a heterozygous state in P26


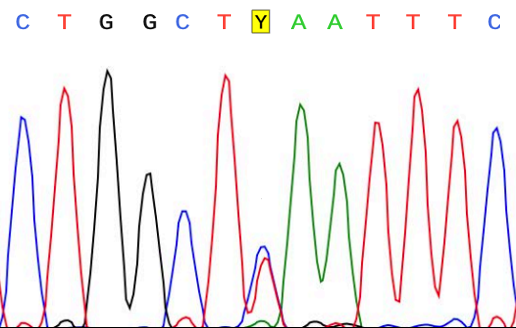


**c.422G>A/p.Arg141His** (chr12:g.18719578C>T)

Identified in a heterozygous state in P9


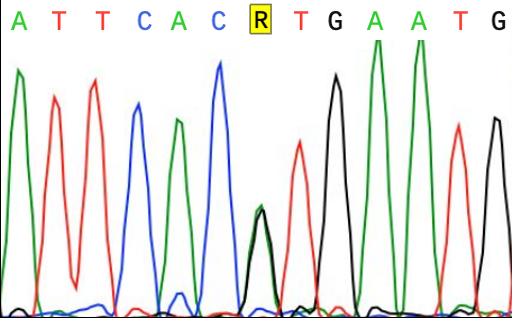


**c.698A>T/p.His233Leu** (chr12:g.18712858T>A)

Identified in a heterozygous state in P2, P21, P34, P35 and P44 (also in brother of P44)


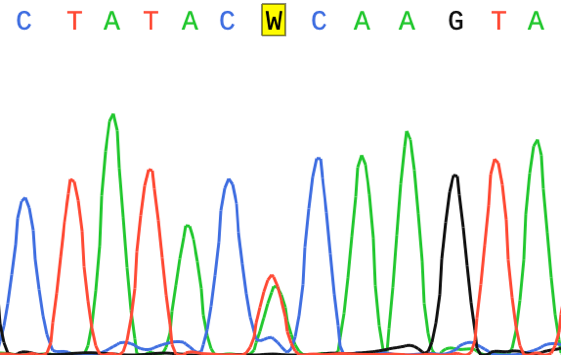


**c.964A>T/p.Lys322*** (chr12:g.18701554T>A)

Identified in a heterozygous state in P2


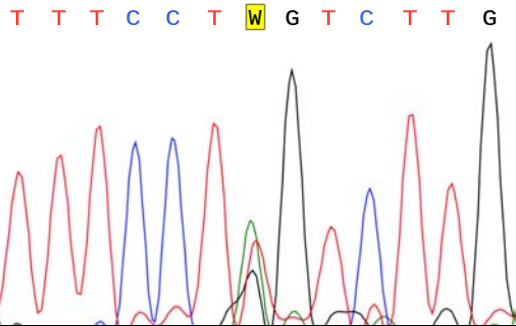


**c.1136T>C/p.Ile379Thr** (chr12:g.18699832A>G)

Identified in a heterozygous state in P36


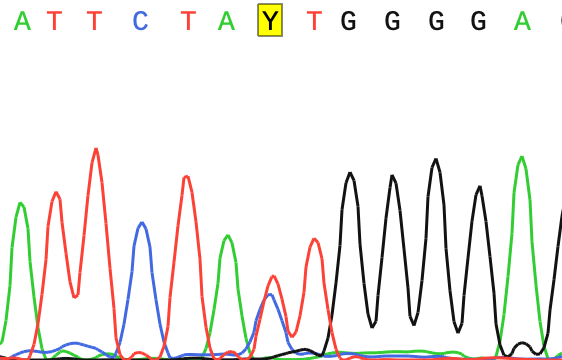


**c.1499C>T/p.Ser500Leu** (chr12:g.18688181G>A)

Identified in a heterozygous state in P3, P4, P15, P26 Identified in a homozygous state in P7, P18, P20, P23


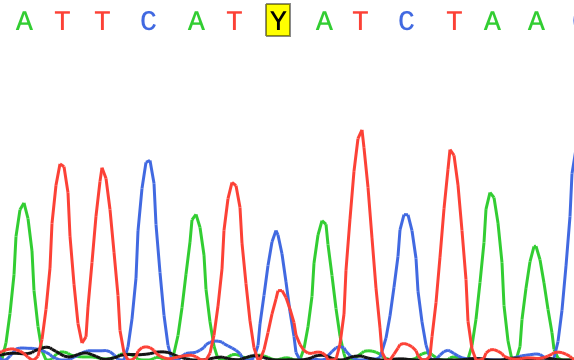

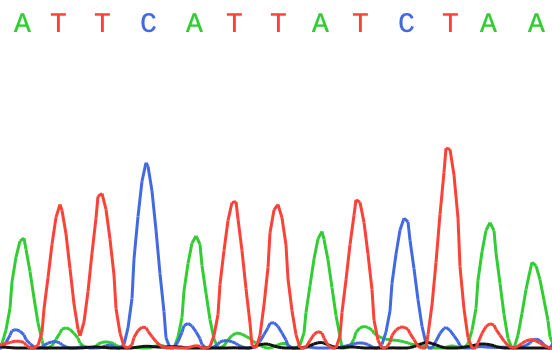


***ACTL9* variants**

**c.812G>C/p.Arg271Pro** (chr19:g.8697890C>G)

Identified in a heterozygous state in P34


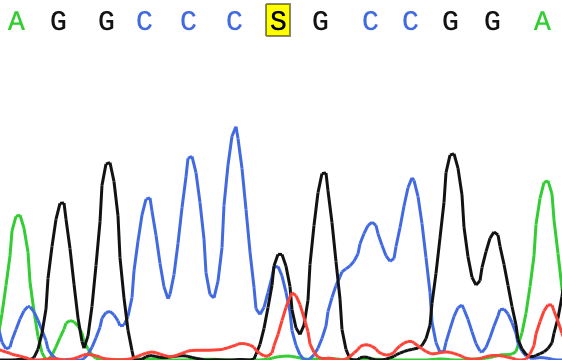


***ACTL7A* variants**

**c.547T>C/p.Tyr183His** (chr9:g.108862869T>C)

Identified in a heterozygous state in P32


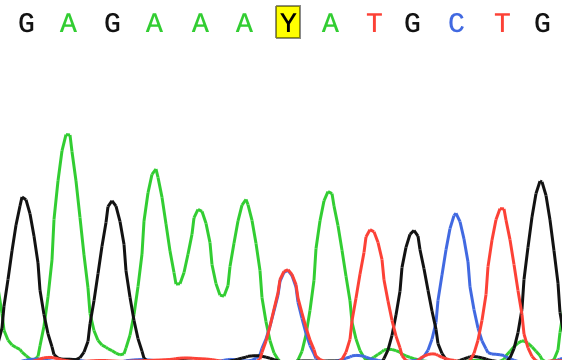


**c.640G>A/p.Gly214Ser** (chr9:g.108862962G>A)

Identified in a homozygous state in P46


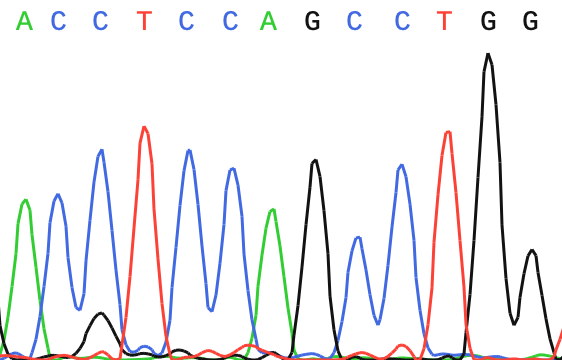


**c.657G>A/p.Val219=** (chr9:g.108862979G>A)

Identified in a heterozygous state in P54


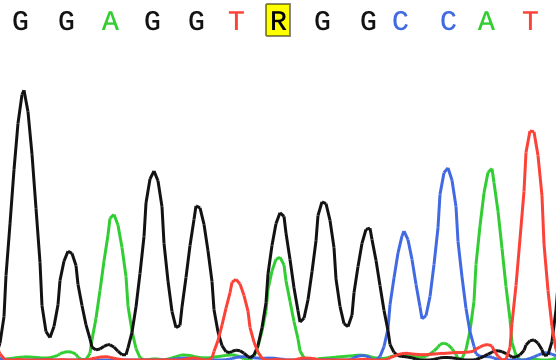


**c.1018G>A/p.Val340Met** (chr9:g.108863340G>A)

Identified in a heterozygous state in P54


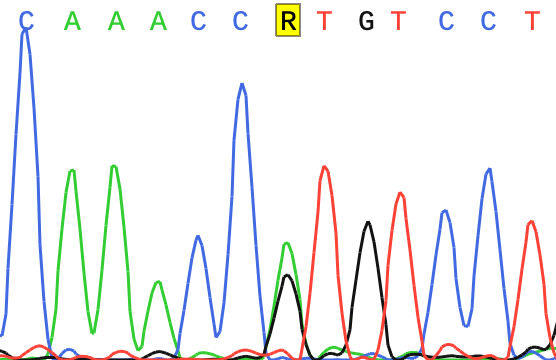


**c.1088dup/p.Ser364Glnfs*9** (chr9:g.108863410dup)

Identified in a homozygous state in P29


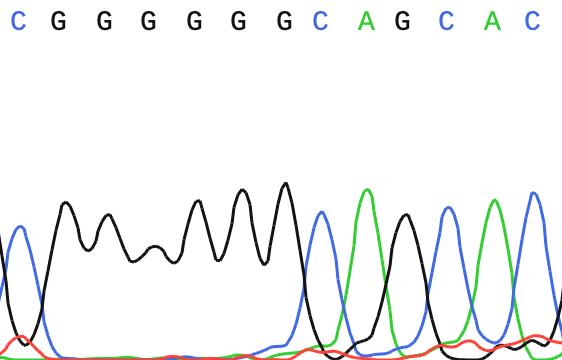


**c.1117C>T/p.Arg373Cys** (chr9:g.108863439C>T)

Identified in a homozygous state in P46


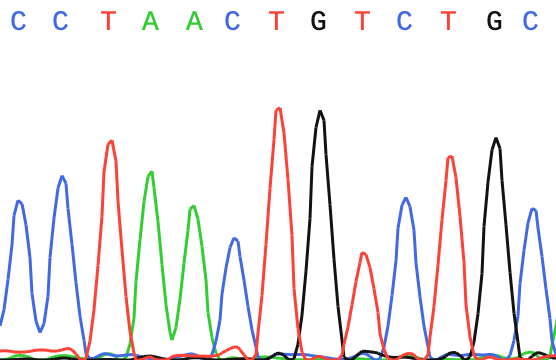


**Supplementary Figure S2 Sanger sequencing confirmation of identified variants in *PLCZ1, ACTL7A,* and *ACTL9* genes.**


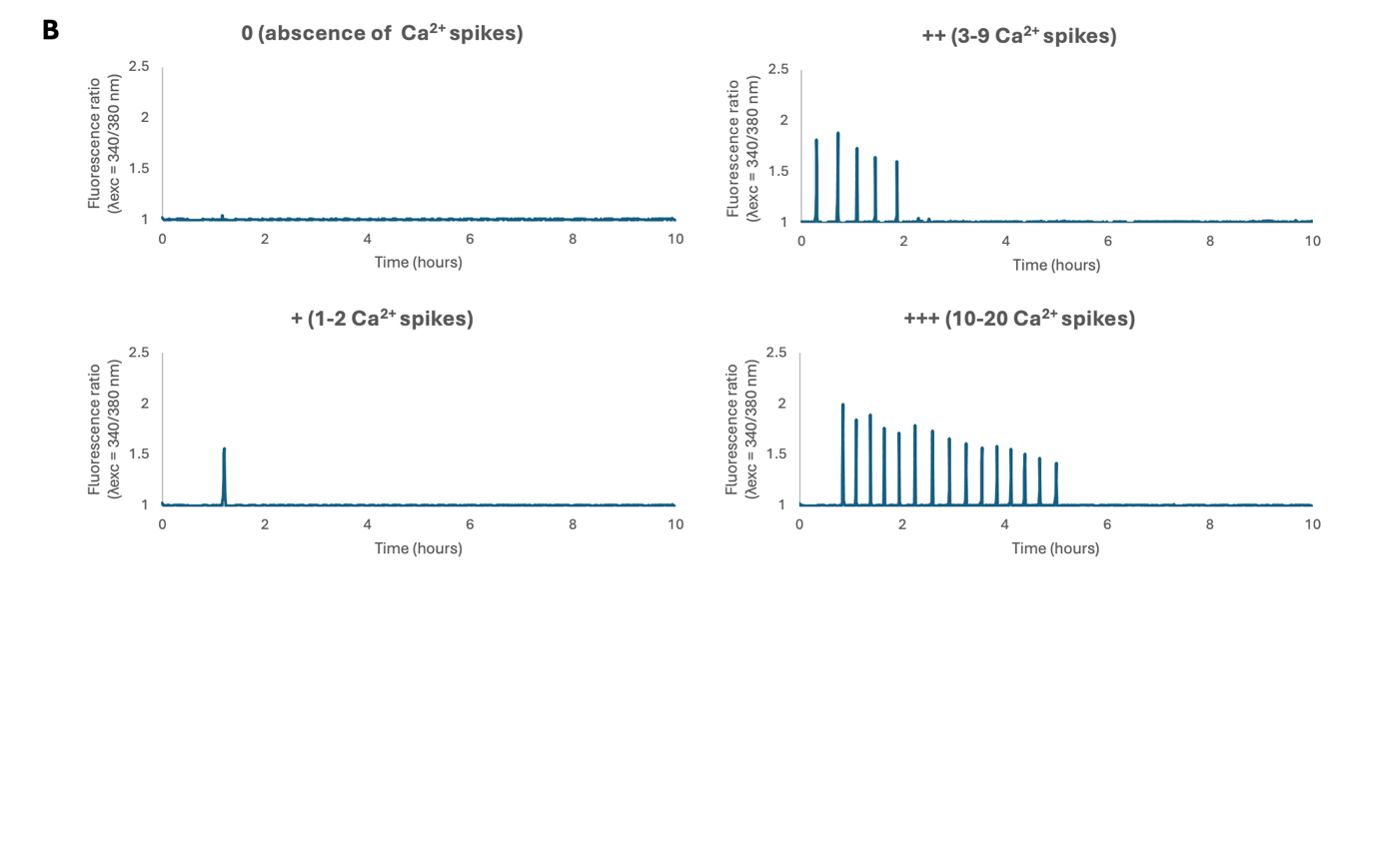

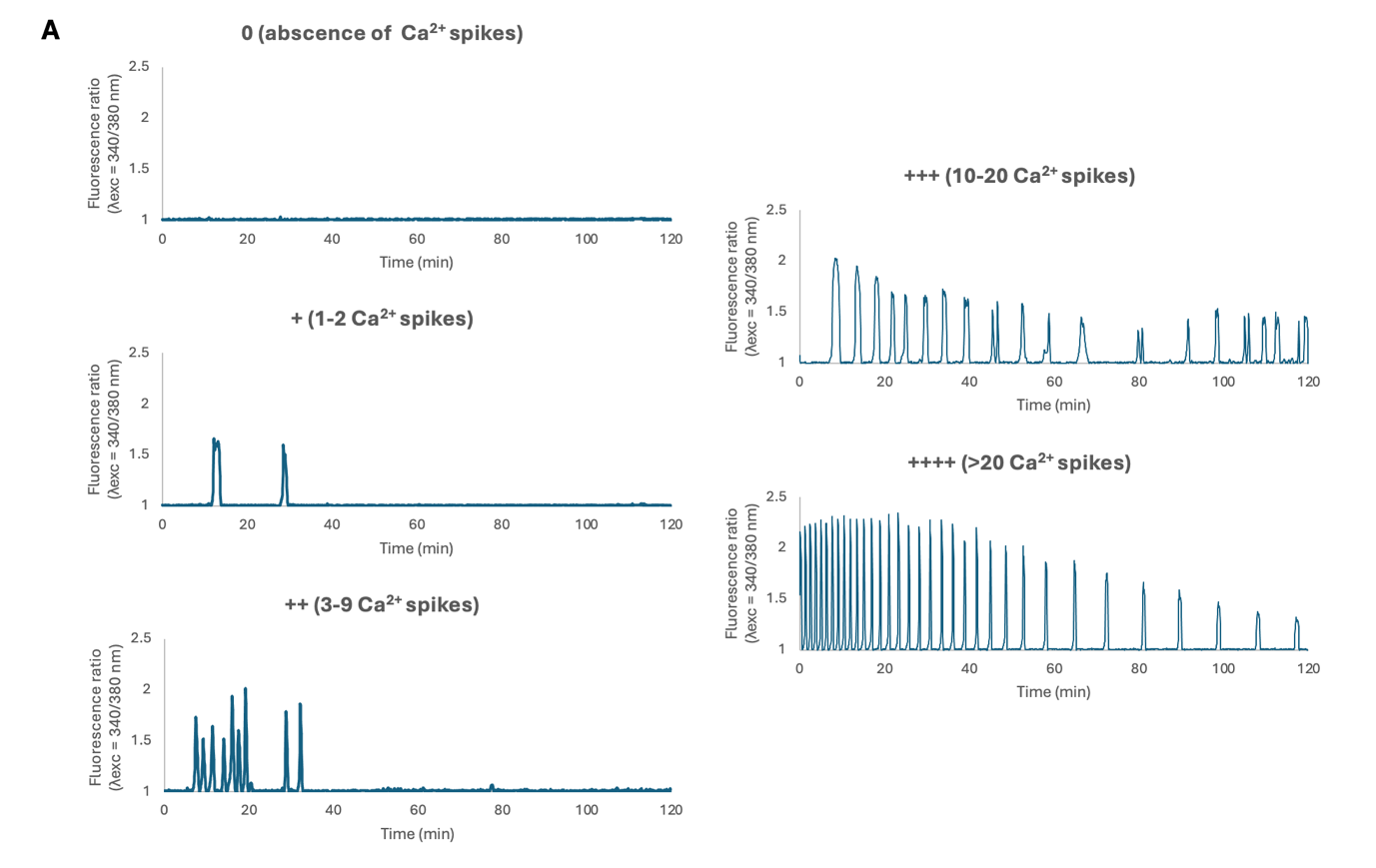


**Supplementary Figure S3 Representative Ca^2+^ oscillation patterns observed per injected oocyte.** A) During mouse oocyte calcium analysis (MOCA) in a 2 h measuring period B) During human oocyte calcium analysis (HOCA) in a 10 h measuring period. Ca^2+^ spikes frequencies are classified as follows: (0) absence of spikes, (+) 1-2 spikes, (++) 3-9 spikes, (+++) 10-20 spikes, or (++++) >20 spikes. λexc: excitation wavelength.

**
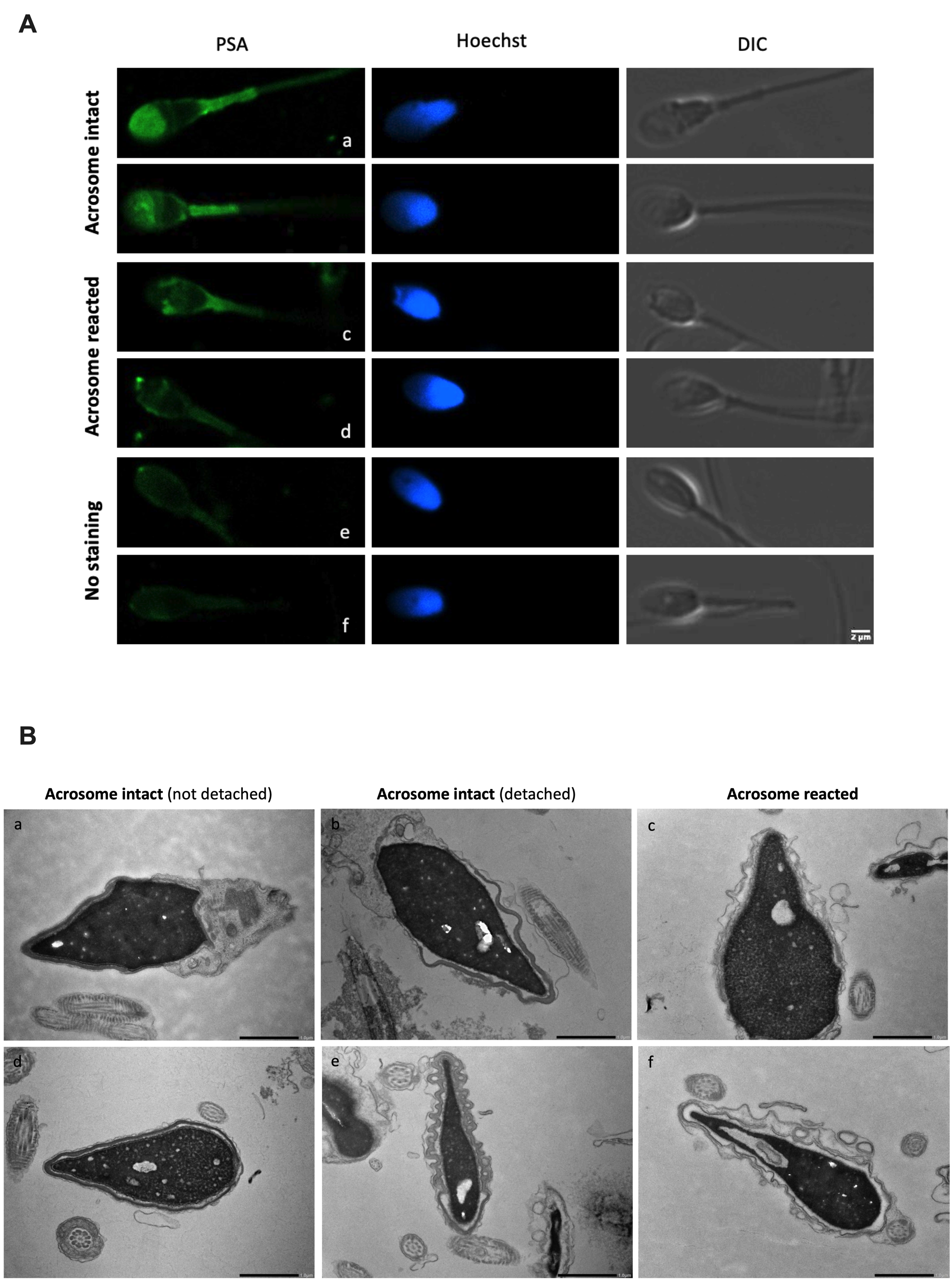
**

**Supplementary Figure S4 Acrosome analysis.** A) Immunofluorescence of sperm acrosome structure using PSA-FITC (green). DNA is stained with Hoechst (blue). DIC: differential interference contrast. a,d) Representative images of intact acrosome. c,d) Representative images of reacted acrosome. e,d) Representative images of non-stained acrosome. Scale bar = 2 μm. B) Sperm head ultrastructure morphology observed during TEM analysis at 10000x. Acrosomes were considered detached from the nuclear envelope when the distance of detachment was ≥50% of the size of the acrosome or showed a clear folding and curved shape, with more than 50% of the observed acrosome detached. a,d) Representative images of intact acrosome not detached from the nuclear envelope. b, e) Representative images of intact acrosome detached from the nuclear envelope. c, f) Representative images of reacted acrosome. Scale bar = 1 μm.
